# Treatment of sugarcane vinasse in AnMBR and UASB: process performance and microbial community comparison

**DOI:** 10.3389/fbioe.2024.1489807

**Published:** 2024-11-08

**Authors:** Beatriz Egerland Bueno, Victor S. Garcia Rea, Flávia Talarico Saia, Gustavo Bueno Gregoracci, Gustavo Dacanal, J. B. van Lier, Marcelo Zaiat

**Affiliations:** ^1^ Biological Processes Laboratory, Department of Environmental Engineering, University of Sao Paulo, São Carlos, Sao Paulo, Brazil; ^2^ Sanitary Engineering section, Department of Water Management, Delft University of Technology, Delft, Netherlands; ^3^ Institute of Marine Sciences, Federal University of Sao Paulo, Santos, Sao Paulo, Brazil; ^4^ Department of Food Engineering, University of Sao Paulo, Pirassununga, Sao Paulo, Brazil

**Keywords:** anaerobic digestion, anaerobic membrane bioreactor, ultrafiltration, sugarcane vinasse, sludge retention, microbial community

## Abstract

Vinasse is a by-product of sugarcane processing which is often used in fertigation; however, the direct use of vinasse harms the environment and reduces soil productivity due to its physicochemical properties. Anaerobic digestion (AD) offers an alternative to mitigate part of the negative effects. Anaerobic high-rate reactors, which mainly rely on sludge granulation, are mostly used in AD of vinasse wastewater. However, the composition of vinasse such as high concentration of solids and organic matter, high salinity, low pH, and high concentrations of sulfate, affect granule formation, leading to sludge washout. Anaerobic membrane bioreactors (AnMBR) present an alternative for vinasse treatment, eliminating the need for sludge granulation and producing a nutrient-rich effluent with minimal residual organics and no suspended solids. Research on sugarcane vinasse treatment using AnMBRs is limited. Most studies have employed submerged internal membrane modules, highlighting the need for further research with different reactor configurations to enhance process performance. In this study, an AnMBR equipped with an external inside-out crossflow ultrafiltration membrane was compared to an upflow anaerobic sludge blanket (UASB) reactor for the treatment of sugarcane vinasse. At a volumetric organic loading rate of up to 6 g COD. L^-1^.d^-1^, the UASB reactor reached 75% ± 7% of COD removal efficiency whereas the AnMBR generated a solids-free effluent and reached 88% ± 2% of COD removal efficiency. Microorganisms such as *Clostridia, Bacteroidia, Mesotaga, Syner-01, Dehalococcoidia*, *Bacteroidia*-DMER64, and *Methanolinea* were found as the most abundant. The results highlight the AnMBR potential as an effective alternative for treating sugarcane vinasse while overcoming the challenges posed by unsatisfactory sludge granulation.

## 1 Introduction

Vinasse is the main by-product generated during ethanol production from sugarcane. Currently, fertigation is the main use of it (Fuess and Garcia, 2014). In addition to prevent its release into water bodies, fertigation offers other benefits such as the recovery of nutrients present in the vinasse. Vinasse has the potential to increase the soil’s biological activity and improve its physical structure if it is properly disposed (Van Haandel, 2005). Nevertheless, vinasse has a high concentration of solids, organic matter, sulfate, and low pH, which can compromise the productive quality of soils (Fuess and Garcia, 2014). Therefore, its direct application in fertigation becomes a residual management problem, making the treatment of vinasse essential for the environmental sustainability of sugarcane distilleries (Fuess and Garcia, 2014).

Anaerobic digestion (AD) is an alternative for the simultaneous degradation of organic pollutants and energy recovery through biogas generation. The use of methane present in biogas might increase the energy efficiency of the mills producing sugar and ethanol (Van Haandel and van Lier, 2015). The application of AD for the treatment of industrial wastewater has shown remarkable progress in the past 2 decades due to the development and improvement of high-rate anaerobic reactors (Van Lier et al., 2020). Despite the progress, further research is still needed to overcome the limitations of available reactors and increase the efficiency of organic matter conversion and, therewith, the energy recovery through methane generation.

Commonly, high-rate anaerobic reactors make use of granular sludge (Van Lier et al., 2020). However, the granules tend to disintegrate and are washed out of the reactors during treatment of effluents containing toxic or inhibitory compounds, with high concentrations of suspended and dissolved solids, or at high temperature (Muñoz Sierra et al., 2019). In such cases, the treatment process must be based on the retention of sludge by other means (Dereli et al., 2012).

Sludge retention is achieved using anaerobic membrane bioreactors (AnMBR). The effective sludge retention provides ideal conditions for organic matter degradation and generates a final effluent without suspended solids. Therefore, the effluent obtained from AnMBRs is of higher quality in comparison to the effluents of other anaerobic processes, allowing the possibility of water reclamation (Ozgun et al., 2013). Muñoz Sierra et al., 2019 compared the performance of an upflow anaerobic sludge blanket (UASB) reactor and an AnMBR for the treatment of phenolic and saline wastewater. The AnMBR exhibited greater stability under increasing concentrations of phenol up to 5 g.L^−1^ and salinity up to 26 g Na^+^.L^−1^. Sludge washout was observed in the UASB reactor at same concentrations of phenol and sodium as the AnMBR, leading to a severe loss of COD removal efficiency in the UASB reactor.

Several configurations of anaerobic reactors have been used in the treatment of sugarcane vinasse: anaerobic sequencing batch reactor with immobilized sludge and with mechanical agitation (AnSBBR) (Albanez et al., 2016), structured-bed reactor (FVR) and packed-bed reactors (PBR) (Aquino et al., 2017), two-phases reactor (Fuess et al., 2018), and thermophilic bed reactors (Santos et al., 2014). However, studies evaluating AnMBR for vinasse treatment are still scarce in the literature. Mota et al. (2013) and Santos et al. (2017) investigated the treatment of sugarcane vinasse with the use of a two-stage submerged anaerobic bioreactor (2S-AnMBR) comprised by an acidogenic reactor followed by a methanogenic reactor with a polyetherimide hollow fiber-type submerged membrane. Both studies showed high removal efficiencies of COD (≈97%) and sulfate (≈87%). However, problems with membrane fouling were reported affecting the filtration performance. The membrane’s cleaning in place (CIP) is less practical in such AnMBR configuration as the membranes need to be removed from the reactor, disturbing the system until the cleaning is completed. These procedures may affect the process and increase the costs in full-scale installations.

Magalhães et al., 2020 compared ultrafiltration and reverse osmose membranes for polishing sugarcane vinasse after biological treatment using 2S-AnMBR with a submerged membrane. The raw sugarcane vinasse was pre-treated by ultrafiltration (UF) followed by the 2S-AnMBR. The UF was applied to reduce the typical vinasse organic load and suspended solids fluctuation, and consequently, to prevent membrane fouling in the 2S-AnMBR. After anaerobic treatment, the 2S-AnMBR permeate was utilized as the feed solution for the experiments with nanofiltration and reverse osmosis membranes. The integrated UF-2S-AnMBR process applied for the sugarcane vinasse treatment was efficient in removing organic matter, achieving 93.3% COD removal efficiency. However, the application of filtration steps before the biological treatment would be unfeasible on an industrial scale due to the large volumes produced and the high levels of solids present in the vinasse (Magalhães et al., 2020).

To date, no studies in the literature have utilized an AnMBR with an external membrane module for vinasse treatment. The AnMBR equipped with an external membrane module offers some advantages over a submerged module despite being an energy-intensive process due the need to maintain a high cross-flow velocity (CFV) to prevent membrane fouling and ensure stable filtration. These advantages are, for example, easier hydrodynamic control, membrane cleaning (CIP) and replacement avoiding the exposure of the anaerobic sludge to aerobic conditions (Le-Clech et al., 2006; Lin et al., 2013; Smith et al., 2012).

In the present study, a UASB reactor and a single stage methanogenic AnMBR equipped with an inside-out external crossflow UF membrane module were used for the treatment of sugarcane vinasse. The treatment performance was compared in terms of organic loading rate and COD removal efficiency. Additionally, the sludge from both reactors was characterized molecularly (*16S rRNA* gene) during their operational period, providing insights into the changes in the microbial community occurring within the different operation stages.

## 2 Materials and methods

### 2.1 Characterization of the sugarcane vinasse and inoculum

Sugarcane vinasse was collected at São Martinho Plant, Pradópolis, São Paulo, Brazil. The samples were stored in plastic containers and frozen at −20°C until their use. A chemical and physicochemical characterization of the vinasse is shown in Table 1. The anaerobic sludge used to seed the reactors was collected from a mesophilic UASB reactor treating poultry wastewater from the poultry farm Dacar S.A., located in Tietê, São Paulo, Brazil. The seed sludge was granular and had a TSS concentration of 28.0 ± 0.7 g.L^−1^ and a VSS concentration of 24.4 ± 0.2 g.L^−1^.

**TABLE 1:** Chemical and physicochemical characterization of the sugarcane vinasse.

Acetone	11 mg. L^-1^
Acetic acid	415 mg. L^-1^
Calcium	1,400 mg. L^-1^
Carbohydrates	3,750 mg. L^-1^
Chlorides	2,800 mg. L^-1^
COD total	33700 mg. L^-1^
COD soluble	20680 mg. L^-1^
Ethanol	480 mg. L^-1^
Latic acid	720 mg. L^-1^
Magnesium total	505 mg. L^-1^
Manganese total	2.8 mg. L^-1^
Ammoniacal nitrogen	92 mg. L^-1^
Nitrate nitrogen	430 mg. L^-1^
Nitrogen total	1,030 mg. L^-1^
Potassium	2,840 mg. L^-1^
Polyphenols	2,450 mg. L^-1^
Phenol	2.0 mg. L^-1^
Phosphate total	180 mg. L^-1^
Phosphorous	60 mg. L^-1^
Propionic acid	16.1 mg. L^-1^
Sulfate	1950 mg. L^-1^
Electric conductivity	9,925 μS cm^-1^
pH	4.5
Total Solids	53.43 ± 1.3 g. L^-1^
Total Volatile Solids	21.9 ± 0.24 g. L^-1^
Total Suspended Solids	9.11 ± 1.4 g. L^-1^
Volatile Suspended Solids	7.06 ± 0.1 g. L^-1^

### 2.2 Reactors’ operational conditions

**FIGURE 1:**
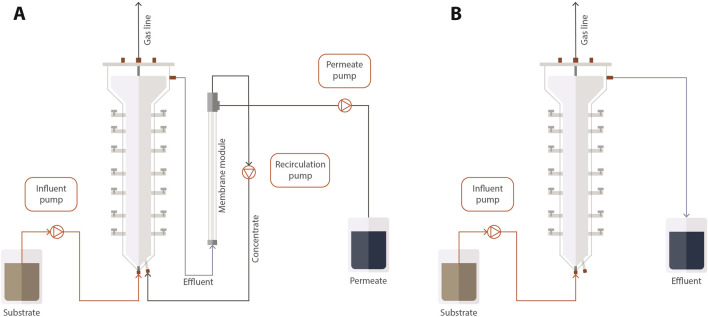
Scheme of the AnMBR **(A)** and the UASB reactor **(B)**.

Both reactors were built in acrylic and had a total volume of 4.0 L and a working volume of 3.5 L. Reactor 1 (AnMBR) comprised a UASB reactor coupled to an external module with a tubular-ultrafiltration PVDF membrane (Pentair X-Flow, Netherlands) (Figure 1). The membrane had a diameter of 5.2 mm, length of 0.64 m, and a nominal pore size of 30 nm, resulting in a surface area of 0.00105 m^2^. Approximately, one-third of the reactors’ working volume was filled with seed sludge resulting in a VSS concentration of 8 ± 0.5 gVSS.L^−1^. The recirculation flow through the external membrane module was 432 L d^-1^. A crossflow velocity of approximately 0.25 m s^−1^ was applied for reducing membrane fouling by creating shear force on the membrane surface, which helps prevent the accumulation of solids. In addition, a relaxation period was used to prevent the membrane fouling. Reactor 2 was a UASB reactor operated at the same conditions as the AnMBR. The reactors were kept in a chamber at 37°C. The pH was maintained at 7.0. The pH correction in the feeding solution was performed by adding sodium bicarbonate to the substrate in the proportion of approximately 0.3 g NaHCO_3_.g^−1^COD_fed_ according to Aquino et al. (2017). The operation of the reactors (230 d) was divided into six phases (I to VI) (Table 2). The operation started with the adaptation of the sludge to tap water-diluted vinasse (CODt = 2 g.L^−1^). The initial HRT was 3 d, resulting in an OLR of 0.6 gCOD. L^-1^. d^−1^ and a membrane flux (J) of 6 L m^-2^. h^−1^ in the case of the AnMBR. The diluted vinasse was used from Phase I to Phase IV. The OLR was gradually increased until no dilution was applied, when the reactors showed a stable COD removal efficiency higher than 85% (Aquino et al., 2017). The reactor was fed with non-diluted vinasse in Phases V and VI.

**TABLE 2:** Conditions in the reactors’ operation phases.

Parameter	Unit	Phases					
		I	II	III	IV	V	VI
OLR	g COD.L^-1^. d^-1^	0.6	1.3	2.6	3	6	10
J^a^	L.m^-2^.h^-1^	6	6	6	3	3	6
HRT	d	3	3	3	6	6	3
COD feed	g COD.L^-1^	2	4	8	18	35	30
COD added	g COD.d^-1^	2.1	4.5	9	10.5	21	35
COD/SO_4_^2-^	g.g^-1^	15	15	15	16	10	12
Duration	d	40	60	40	40	30	30

^a^ In the AnMBR.

### 2.3 Physicochemical analyses

Parameters such as pH, volatile fatty acids (VFA) (Adorno et al., 2014), total COD (CODt), soluble COD (CODs), and carbohydrates (CH) (APHA, 2017) were analyzed 3 to 5 times a week. Sulfate and phenol removal were evaluated according to APHA (2017). The samples collected from the UASB reactor were filtered through 0.45 μm membranes prior to the measurement of CODs, CH, and VFA. It was not necessary to perform an extra filtration step in the effluent of the AnMBR (permeate) as it already passed through the UF membrane (nominal pore size <0.45 μm). Therefore, the reactors were compared in terms of CODt. The biogas composition (methane, hydrogen, and carbon dioxide) was analyzed by a gas chromatograph (model GC-2010, Shimadzu) equipped with a thermal conductivity detector (GC/TCD) and a Carboxen 1010 PLOT column (30 m, 0.53 mm) according to Araujo et al. (2023). The results are reported as average ±standard deviation.

### 2.4 Particle size distribution

Sludge samples were collected from inside the reactors for particle size distribution (PSD) analysis. Samples containing approximately 1 g of sludge were deposited on a glass slide and observed under a stereomicroscope (Stereo Discovery V8, Zeiss, Jena, Germany) equipped with a digital camera (Axiocam ICC 3, Zeiss). The stereomicroscope captured the two-dimensional (2D) silhouettes of the particles. The binary image filtering and analysis of the individual particles were performed using ImageJ v1.60 software (National Institutes of Health, United States). The total number of particles $\left(N_{i}\right)$ was at least 500, by capturing up to 50 microscopic images for each sample. The 2D area $\left(A_{p}\right)$ was used to calculate the individual particle diameters (*d*), represented by Equation 1 (Dacanal et al., 2016; Russ, 2011). Histograms were computed to evaluate the particle size distribution which visually evidenced the granular or dispersed sludge states over time.

$$d_{i}=\sqrt{\frac{4{\;A}_{p\_i}}{\pi }}$$ (1)

### 2.5 16S rRNA gene amplicon sequencing and bioinformatics

Sludge of both reactors was sampled at the end of each operational phase. Moreover, the sludge attached to the AnMBR’s membrane was sampled at the end of the operational Phases III, V, and VI. The sludge samples were stored in 2 mL Eppendorf, washed with PBS-buffer (PBS: 0.13 M NaCl, 7 mM Na_2_HPO_4_, 3 mM Na_2_H_2_PO_4_, pH 7.2) and centrifuged for 10 min at 6,000 rpm on an Eppendorf Centrifuge 5417R at room temperature. The resulting pellet (∼1 g) was collected and kept at −20°C until the DNA extraction was performed.

Genomic DNA extraction was performed using the FastDNA SPIN kit for soil (MP Biomedicals, Irvine, CA, United States). Subsequently, the 16S rRNA gene was amplified using the primer sets V3–V4 (341F/785R) described in Klindworth et al. (2012). Primers contained adapter overhangs according to manufacturer’s recommendations (Illumina manual). Paired-end sequencing of the final library was conduct on the MiSeqTM System (Illumina, Inc., San Diego, CA) with a v3 reagent kit (600 cycles) according to the manufacturer’s guidelines. All library preparation and sequencing were performed as a service from the NGS Soluções Genômicas (Piracicaba, Brazil). Sequence analysis was performed in R (R Core Team, 2020) and Linux bash. Primer sequences which are known to interfere in the amplicon sequence variant (ASV) detection were excluded using Trimmomatic (Bolger, Lohse, and Usadel, 2014) which trimmed and removed short sequences as well. Sequences with Ns were removed in DADA2 (Callahan et al., 2016). The DADA2 pipeline was used to predict errors, calculate ASV, and merge pairs. Finally, the merged sequences were annotated using the DECIPHER package against the 16S rRNA genes (version 138.1) (Quast et al., 2013) with calculations performed in R. This process produced 706,762 annotated reads for the 16 samples (average ±SD 44,173 ± 14,737) divided into 1,816 ASV and classified into genus level. Stacked column graphs were depicted with GraphPad Prism 8.

A regularized canonical correlation analysis (rCCA) was applied using ASV and environmental parameters with the R package mixOmics (Cao et al., 2016) to address more directly how environmental parameters and individual ASV were related. The rCCA was applied separately in the samples of each reactor and only ASVs with total abundance over 2,500 were included; the same inoculum was considered in both analyses. Samples were analyzed at the ASV level, retaining unclassified reads. Graphs were produced in Graphpad 8.0.

Finally, diversity analysis and PCA plots were produced in R, using the “vegan” and “ape” packages (Oksanen et al., 2022). Diversity employed the Shannon Neff correction while richness was calculated after rarefication to the lowest count. ASV data was transformed to Hellinger and the 15 most abundant ASVs were included in the Bray-curtis distance matrix for the PCoA plot. The sequences were deposited in the SRA (NCBI) database under the accession number PRJNA1149308.

### 2.6 Other statistical analyses

Student’s t-test was applied with a 95% confidence level using the Statistica 13.4.0.14^®^ software (TIBCO Software Inc., United States) to compare the COD, CH, sulfate and polyphenols removal efficiencies values obtained in triplicates during the monitoring of both reactors.

## 3 Results and discussion

### 3.1 Performance of the anaerobic reactors

The assessment of the performance of the AnMBR and the UASB reactor is presented in the coming (sub) sections. The examined parameters include COD, carbohydrate, sulfate reduction, and polyphenol removal efficiencies. The reactors were operated under varying OLR and HRT providing insights into their efficiency, resilience to operational stress, and membrane fouling dynamics over six distinct phases of operation.

Table 3 shows the different parameters and values obtained during the operation of both reactors.

**TABLE 3:** Operational parameters and reactors’ performance during the different phases of the study.

Parameters	AnMBR						UASB					
Phases	I	II	III	IV	V	VI	I	II	III	IV	V	VI
SLR (g COD gVSS^-1^ d^-1^)	0.1	0.31	0.42	0.26	0.33	0.4	0.1	0.1	0.14	0.18	0.34	0.18
RE_CODt (_%)	85 ± 8	88 ± 2	85 ± 4	89 ± 3	88 ± 2	37 ± 4	81 ± 9	82 ± 6	76 ± 13	85 ± 2.3	75 ± 7	37 ± 14
RE_CH_ (%)	96 ± 1.5	94 ± 2	92 ± 5	92 ± 2	94 ± 2	88.5 ± 2	95 ± 1.2	93 ± 2	80 ± 11	91 ± 1	87 ± 3	79 ± 6
RE_sulfate_ (%)	—	81 ± 22	91 ± 6	98 ± 1.5	99 ± 0.6	99 ± 0.6	—	73 ± 24	77 ± 20	98 ± 3	99 ± 0.5	99 ± 2
RE_phenol (_%)	—	—	41 ± 2	61 ± 11.5	58 ± 15	30 ± 17	—	—	15 ± 11	41 ± 14	11 ± 2	0
CH_4_ (%)	83 ± 16	65 ± 15	56 ± 11	51 ± 9	50.7 ± 9	20 ± 9	82 ± 21	55 ± 16	48 ± 4	42 ± 6	58 ± 11	35 ± 17

Values shown as average ± standard deviation. RE, removal efficiency.

#### 3.1.1 COD removal

**FIGURE 2:**
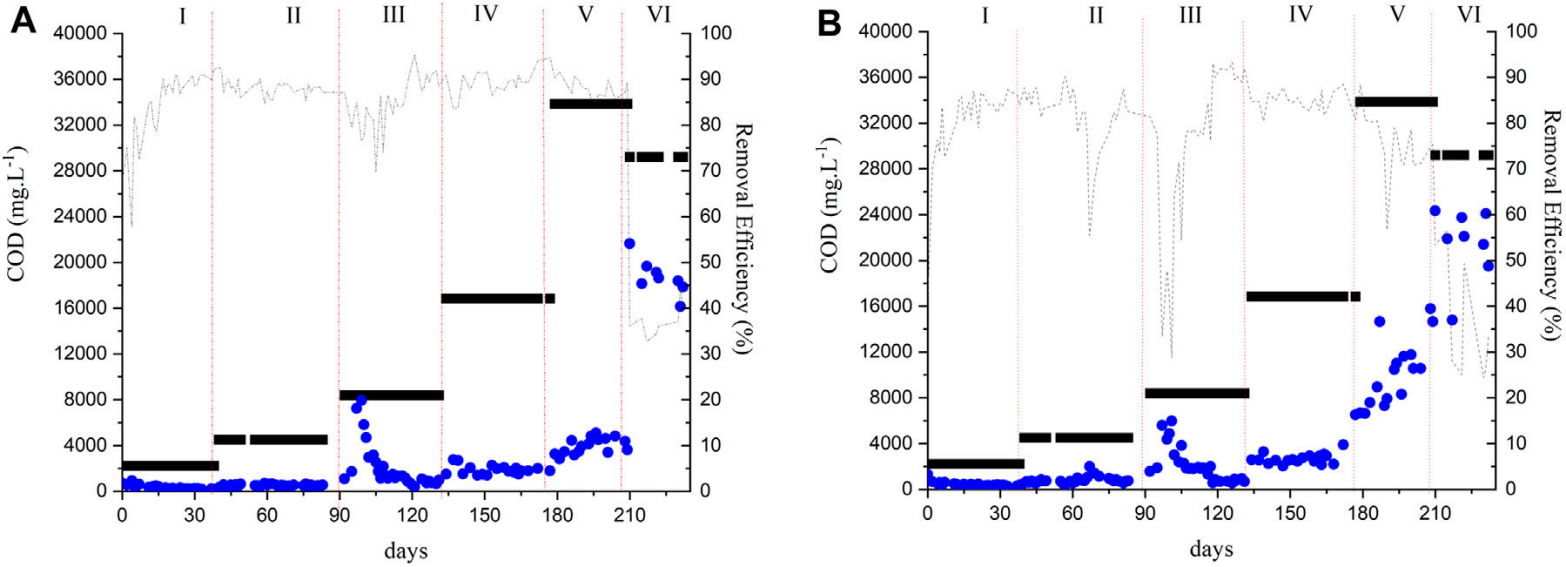
Reactors monitoring over time of COD removal **(A)** AnMBR **(B)** UASB: (■) Feed (●) Effluent (---) Removal efficiency.

In Phase I, the sludge of both reactors was adapted to the vinasse for approximately 40 days with an OLR of 0.6 g COD.L^−1^.d^−1^ corresponding to a sludge loading rate (SLR) of 0.1 gCOD.gVSS^-1^.d^−1^. The OLR was gradually increased after the COD removal efficiency was stable (85%). The HRT was 72 h in the three initial phases corresponding to a membrane flux of 6 L m^-2^. h^-1^. Figures 2A, B show the COD removal efficiencies of the AnMBR and the UASB reactor, respectively, during the operational phases. Results are shown as CODt (Section 2.3).

The COD removal efficiencies of the AnMBR and the UASB in Phase I had an average of 85% ± 8% and 81% ± 9%, respectively. These values did not differ statistically (*p* > 0.05) indicating the reactor configuration did not affect the removal efficiency during the adaptation period. VFAs were detected in the effluent of both reactors in concentrations lower than 0.15 g. L^−1^ (Supplementary Figure S1). In Phase II, the AnMBR and the UASB reactors achieved 88% ± 2% and 82% ± 6% of COD removal efficiency, respectively. The removal efficiencies differed statistically (*p* < 0.05) between the two reactors, indicating a better performance of the AnMBR over the UASB reactor in terms of COD removal efficiency. In Phase II, no VFA were found in the effluent of both reactors.

The COD removal efficiency of both reactors decreased after increasing the OLR in Phase III. The UASB reactor showed a higher decrease in the COD removal efficiency with an average of 76% ± 13% whereas the AnMBR had an average of 85% ± 4.5%. The COD removal efficiencies differed statistically (*p* < 0.05). A higher VFA concentration of mainly acetate and propionate was observed in the AnMBR’s permeate in the beginning of Phase III in comparison to the UASB reactor’s effluent. This fact may be related to the increased OLR, causing an overloading of the reactor. During Phase III, severe membrane fouling was observed. Even after physical and chemical cleaning procedures, the membrane was no longer functioning properly. The membrane module was then replaced for a new module at the end of Phase III. The observed irreversible fouling was attributed to several reasons, for example, inorganic precipitations due to high Ca^2+^ concentrations, high suspended solids concentrations (TSS concentration in the reactor was 12.8 g. L^-1^ in Phase III), and high concentrations of colloidal mater (De Vela, 2021). According to Yee et al. (2019), approximately 93% of the total fouling is composed of organic compounds. However, the presence of inorganic compounds in the wastewater, for example, ammonia, magnesium, calcium, phosphorus, and potassium, can result in the precipitation of inorganic matter such as (potassium-) struvite (KMgPO_4_·6H_2_O/NH_4_MgPO_4_·6H_2_O) and calcium carbonate (CaCO_3_) which may contribute to cake layer compaction and pore blocking (Dvořák et al., 2015). Sugarcane vinasse also has a high concentration of solids and organic matter (Fuess and Garcia, 2014). The presence of cations in the influent and sludge matrix may result in the formation of precipitates in the cake layer. Metal clusters and metal ions in the influent might also a) adsorb to the flocs or biopolymers (ion-biomolecule interactions) through charge neutralization and bridging effect (Wang et al., 2013; Seidel and Elimelech, 2002) and/or b) adsorb on the membranes (ion-membrane interactions) (He et al., 2015) increasing the filtration resistance. Unfortunately, it was not possible to measure the values of transmembrane pressure (TMP) in the present study. The lack of real time TMP information did not allow us to instantly react to fouling event(s). Early amendments to process operation, for example, the application of a higher CFV, or an increased cleaning routine with more intense backwash flows, or longer relaxation periods, might have had prolonged the membrane’s lifetime by decreasing the fouling.

In Phase IV, the flux was decreased to 3 L m^-2^. h^-1^ to reduce the fouling and, consequently, to reduce the membrane cleaning (Galinha et al., 2018). The OLR (3 g COD L^-1^. d^-1^) was kept close to the one applied in Phase III (2.6 g COD L^-1^.d^−1^) and the HRT was increased to 6 d following the decrease in flux. Both reactors had a better performance in Phase IV. However, a significant difference (*p* < 0.05) in the COD removal efficiency was found with values of 89.5% ± 2.9% and 84.6% ± 2.3% for the AnMBR and the UASB reactor, respectively. The VFA concentrations in the effluent of both reactors were low (<0.15 g. L^−1^) during this phase (Supplementary Figure S1).

In Phase V, the reactors had a significant difference (*p* < 0.05) in the COD removal efficiency with values of 88.2% ± 2.15% and 75.5% ± 7.4% for the AnMBR and the UASB reactor, respectively. VFAs were measured in both reactors’ effluent in this phase. Acetate was the main VFA detected in the AnMBR’s permeate reaching values of 0.8 g. L^−1^, indicating instability in methanogenesis. Propionate (1 g. L^-1^) was the main VFA detected in the UASB reactor indicating that the propionate oxidation was impaired. The VFA accumulation could be related to the OLR increase and reactors overload. Aquino et al. (2017) measured COD removal efficiencies of 82% ± 5.3% in the FVR and 86% ± 3.2% in the PBR at a similar OLR (5.5 g COD. L^−1^. d^−1^).

In Phase VI, the OLR was increased to 10 g COD. L^−1^. d^−1^ as the AnMBR had a high COD removal efficiency during Phase V. Similarly, the flux was increased to 6.5 L m^-2^.h^−1^; consequently, the HRT decreased to 3 d. However, the increase in OLR negatively affected both reactors. The COD removal efficiency of the AnMBR and the UASB reactor decreased to 36.3% ± 4.2% and to 33.3 ± 14.5, respectively. High VFA concentrations were detected in the effluent of both reactors. The COD removal efficiency did not differ statistically (*p* > 0.05) between the reactors. Aquino et al. (2017) reported COD removal efficiencies of 75% ± 2% in the FVR and 84% ± 1.3% in the PBR at a similar OLR (10.2 g COD. L^−1^. d^−1^). In another study, Fuess et al. (2018) performed the treatment of sugarcane vinasse in a two-stage reactor (acidogenic reactor followed by a UASB reactor). OLRs of 15, 20, and 25 g COD L^−1^ d^−1^ were applied and COD removal efficiencies of up to 70% were achieved. The imposed changes in the operational parameters in the last phase of the present study may have exceeded the system’s capacity. Even with high removal efficiencies, a gradual increase in OLR would be a better approach to avoid reactor perturbation. Further studies aiming at strategies to control fouling and to achieve higher volumetric organic loads to make the process feasible at large scale are needed.

The percentage of methane in the biogas decreased with the increase in the OLR throughout the phases in both reactors (Table 3). The low methane content in the biogas produced is related to the composition of vinasse (Fuess and Garcia, 2015; Parsaee et al., 2019) which is rich in carbohydrates; therefore, producing a higher CO_2_ content in comparison to more reduced substrates (Moraes et al., 2014).

#### 3.1.2 Carbohydrates removal

In Phases I and II, both reactors had similar CH removal efficiency (Supplementary Figure S2). The efficiencies reached values higher than 90% and no significant statistical difference was found. Carbohydrate conversion was not a limiting factor for the treatment of sugarcane vinasse. Other vinasse components, for example, tannic and humic acids, however, can be more challenging for anaerobic digestion (Fuess and Garcia, 2015; Parsaee et al., 2019). Phase III was the first phase in which the carbohydrate conversion statistically differed (*p* < 0.05) between both reactors. The removal efficiency in the UASB reactor decreased to 80% ± 11% whereas the AnMBR maintained a removal efficiency of 92% ± 5%. In Phase IV, the CH removal efficiency of the AnMBR reached 92.4% ± 2%. The UASB reactor had a better performance in comparison to the previous phase (Phase III), reaching 91% ± 1% of removal efficiency. However, the results were statistically different (*p* < 0.05) between the AnMBR and the UASB reactor. The improvement in CH removal of the UASB reactor might be related to the HRT increase, which allowed better carbohydrate conversion. In Phase V, the AnMBR reached 94% ± 2% CH removal efficiency whereas the UASB reactor reached 87% ± 3%. The results statistically differed (*p* < 0.05). Finally, in Phase VI, the AnMBR and the UASB reactor had CH removal efficiencies of 89.5% ± 2% and 79% ± 6%, respectively, differing statistically (*p* < 0.05).

#### 3.1.3 Sulfate removal

The monitoring of sulfate removal started after Phase II (Supplementary Figure S2). Sulfate removal was observed in both reactors; however, the removal efficiency was variable throughout the operation. In Phase II, the AnMBR reached an average sulfate removal efficiency of 80.6% ± 21.8% whereas the UASB reactor reached 73% ± 24%. In phase III, the mean sulfate removal in AnMBR was 91% ± 6%, being higher than that observed in the UASB reactor with 77% ± 20%. In Phase IV, both reactors had high sulfate removal efficiencies with values of 97.75% ± 1.3% and 97.7% ± 3% for the AnMBR and the UASB reactor, respectively. A similar behavior was observed in Phases V and VI, when both reactors had sulfate removal efficiencies of 99%. No statistical difference (*p* > 0.05) was found for the sulfate removal efficiency during the operation of both reactors. Mota et al. (2013) and Santos et al. (2017) investigated the use of a two-stage submerged anaerobic membrane bioreactor (acidogenic reactor followed by a methanogenic MBR) for the treatment of sugarcane vinasse. In both studies, the sulfate removal efficiency was approximately 87%. Using the same reactor configuration, Silva et al. (2020) evaluated the influence of the COD/SO_4_^2-^ ratio on the performance of the vinasse treatment. The AnMBR showed stable performance in the highest COD/SO_4_^2-^ ratios (50-94), showing high COD (97.5%) and VFA (98%) removal efficiencies, but low removal of sulfate (69.9%). At COD/SO_4_^2−^ ratios close to 10, the opposite was reported with lower COD and VFA removal efficiencies (87% and 69.8% respectively) but high sulfate removal efficiency of 93%. In the present study, sulfate removal efficiency was above 70% for both reactors. The COD/SO_4_^2-^ ratios varied from 10 to 16. The COD removal efficiencies are also in line with the results reported by Silva et al. (2020) at lower COD/SO_4_^2-^ ratio (80%–90%). The increase in sulfate reduction during the operation can be correlated with the increase in the vinasse concentration and, consequently, in the sulfate loading rates in the feed and the establishment of sulfate reducing bacteria (SRB). The increase in SRB activity resulted in sulfide generation and thus H_2_S presence in the biogas. However, H_2_S gas was not possible to be measured by the used GC equipment. In average, total liquid sulfide (TLS) concentrations of 38, 65, and 105 mg.L^−1^ were measured in the UASB reactor, in Phases II, III, and IV, respectively. In the AnMBR, sulfide was measured in concentrations of 28, 60, and 76.7 mg.L^−1^, in Phases II, III and IV, respectively. In Phases V and VI, the TLS concentration increased in both reactors, reaching 130.6 and 128 m.L^−1^ in the AnMBR, and 148.9 and 181 mg.L^−1^ in the UASB reactor, respectively. In agreement with Silva et al. (2020), a COD/SO_4_^2-^ ratio close to 10 resulted in almost complete sulfate reduction. Elemental sulfur can be recovered through the oxidation of sulfide present in the biogas (Camilioti et al., 2016) and subsequently be used as fertilizer together with the other nutrients recovered in the process.

Accumulating sulfide may inhibit bioconversions, but literature provides inconsistent data regarding the inhibitory sulfide levels (Chen et al., 2008). Reported inhibitory sulfide concentrations range from 100 to 800 mgL^−1^ of TLS or roughly 50–400 mgL^−1^ of undissociated H_2_S (Parkin et al., 1990). The values obtained in phases V and VI, especially in the UASB, are higher than the minimum reported values. Thus, some degree of inhibition might had occurred. The competition between SRB and other anaerobic microorganisms influences sulfide concentration in the reactor, however, at COD/SO_4_^2−^ ratios exceeding 10, enough electron donors are available for both sulfate reduction and methanogenesis. Notably, sulfide is harmful to both methanogens and SRB. The presence of sulfate in vinasse impacts the conversion rates of SRB and methanogens at two different levels: i) the competition for electron donors; therefore, reducing the methane production (yield), and ii) inhibition induced by sulfide toxicity to the different microbial groups (Chen et al., 2008).

#### 3.1.4 Polyphenols removal

The monitoring of polyphenols removal began in Phase III (Supplementary Figure S3) when the dilution was stopped. Polyphenols emerge through the breakdown of lignocellulosic structures and during the sugar caramelization upon heating, particularly in evaporation bodies, crystallizers, and centrifuges (Contreras-Contreras et al., 2020; Hoarau et al., 2018). Compounds, such as melanoidins, caramels, and phenolic compounds, as well as humic and tannic acids contribute to the effluent colour and exhibit low biodegradability (Hatano et al., 2008). Moreover, polyphenols are recognized as toxic compounds that interfere with the activity of methanogenic archaea during anaerobic digestion slowing down the kinetic rates of the process, resulting in a reduction of the methane production rates and yield coefficients (Jiménez et al., 2003). The vinasse used in this study had an average polyphenols concentration of 2.450 mg.L^−1^ from which 2 mg.L^−1^ was phenol. The UASB reactor achieved polyphenols removal efficiencies of 20% ± 10%, 55% ± 19%, 20% ± 15% and 0%, in Phases III, IV, V, and VI, respectively. The AnMBR achieved polyphenols removal efficiencies of 44% ± 9%, 70.5% ± 13%, 58% ± 14% and 35% ± 13%, in Phases III, IV, V and VI, respectively), showing a statistically difference (*p* < 0.05) with respect to the UASB reactor results.

Recent studies have shown that the AnMBR has excellent performance in the conversion of phenolic compounds. Garcia Rea et al. (2022) found that the sludge harvested from an AnMBR degrading phenol showed highest phenol conversion rates and was most resistant to the inhibitory effects of other phenolic compounds in comparison to other sludge sources. The authors attributed this to the *in-situ* bioaugmentation of the AnMBR sludge, having a UF membrane as absolute barrier for sludge retention. In a previous study, Muñoz-Sierra et al. (2019) compared the performance of a UASB reactor and an AnMBR treating highly saline phenolic wastewater. The AnMBR exhibited distinctly higher stability than the UASB, which was attributed to its enhanced sludge retention under increasing influent phenol and sodium concentrations. In contrast, complete sludge deflocculation occurred in the UASB reactor, leading to a severe phenol conversion capacity loss.

### 3.2 Particle size distribution and volatile suspended solids

**FIGURE 3:**
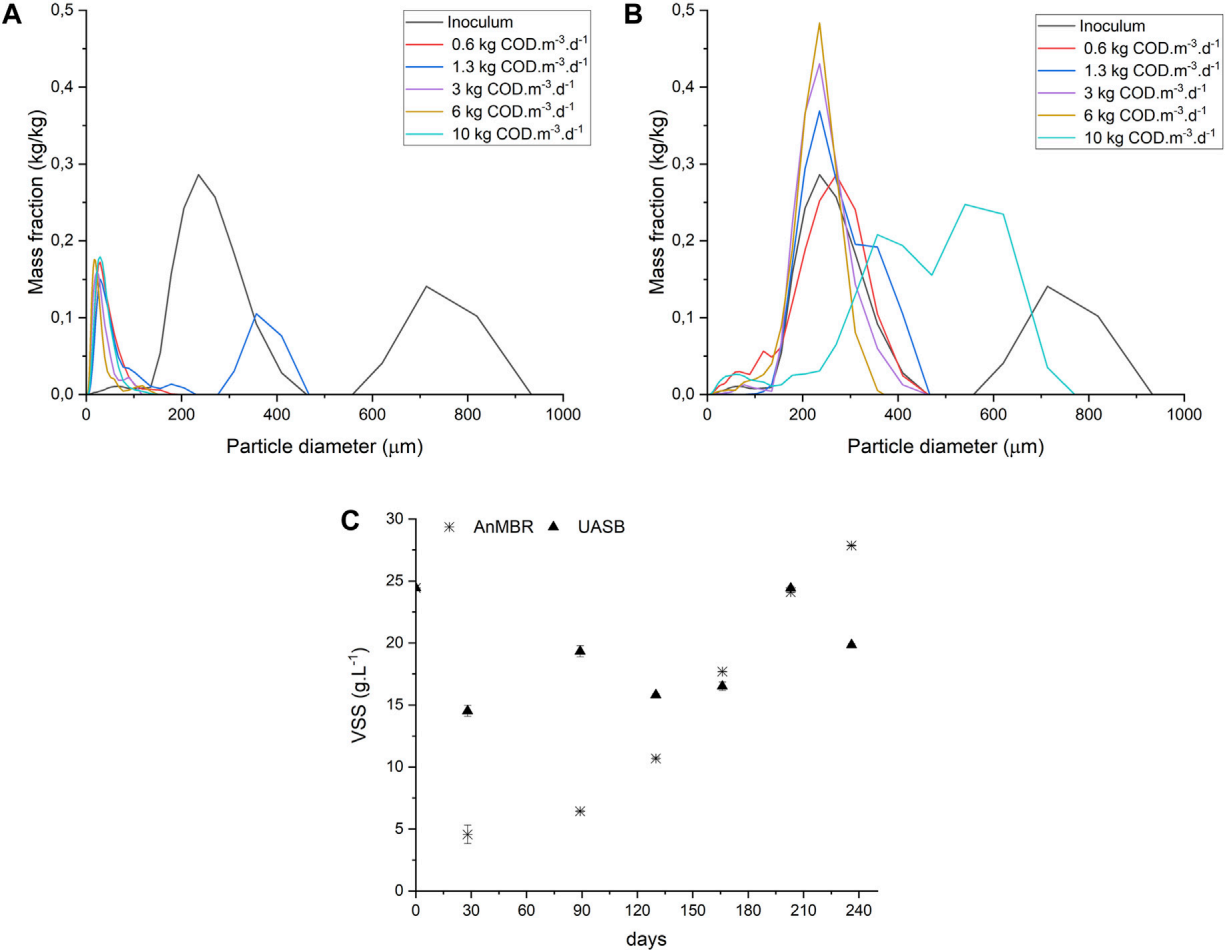
Particle Size Distribution analysis AnMBR **(A)** and UASB **(B)** and Volatile Suspended Solids, n = 3, bars = SD **(C)** through reactors operation.

Particle size distribution analysis was performed on the sludge of both reactors at the end of each phase. In the AnMBR (Figure 3A), the particle size decreased in comparison to the inoculum immediately during Phase I. After this initial decrease, the particle size of the sludge remained constant throughout monitoring. This behavior is expected in a crossflow AnMBR due to the shear forces applied in the membrane module resulting in small-sized particles (De Vela, 2021). The disappearance of the granule structure of the inoculum sludge did not affect process performance parameters such as COD removal efficiency. In the UASB reactor sludge (Figure 3B), the granules’ particle size distribution was similar to the inoculum during almost the entire operation. The distribution became more homogeneous in the stages when the OLR was increased, except in the last phase, in which the trend was divided into two wide peaks.

Figure 3C shows the VSS concentration in the reactors during the experimental period. In the AnMBR, the VSS concentration gradually increased reaching 28 gVSS.L^-1^ at the end of reactor operation. The decreasing particle size did not affect biomass growth. Opposite, the UASB reactor did not show a linear increase in VSS concentration over the operation time. The presence of recalcitrant compounds, high concentration of solids, and high alkalinity are factors that can affect the granule formation process, causing sludge washout during sugarcane vinasse anaerobic treatment (Rajeshwari et al., 2000; van Lier et al., 2020). Granular sludge was often found in the final effluent’s container from the UASB reactor. The average VSS concentration found in the effluent of the UASB reactor was 2.15 ± 0.11 g.L^−1^. Whereas the ultrafiltration membrane modules in the AnMBR provided a high-quality effluent, free of suspended solids.

### 3.3 Microbial community dynamics

#### 3.3.1 Microbial diversity indices of the AnMBR and the UASB reactor

Microbial community samples from the inoculum and at the end of each operational phase of the AnMBR and the UASB reactor, in addition of attached biomass of the AnMBR's membrane, were analyzed through *16S rRNA* gene sequencing. Diversity indicators (Shannon Neff and Richness Indicator, Table 4) showed a quick adaptation of the inoculum to the vinasse in the AnMBR (Phase I) promoting the increase of microbial diversity (Shannon Neff). In the UASB reactor, the adaptation was delayed to Phase II, suggesting the adaptation period in the UASB reactor requires longer period than in the AnMBR. Afterwards, for both reactors, Shannon Neff values decreased across the operational phases until the end of operation, while richness increased, indicating selection of the microorganisms specialized in the anaerobic digestion of the vinasse. Similar adaptation periods were reported for a full scale UASB reactor (Callejas et al., 2022) and a pilot scale HAnBR (Pierangeli et al., 2024) treating sugarcane vinasse. The biomass attached to the membrane of the AnMBR was harvested during fouling episodes. Its microbial analysis showed lower diversity and richness indices than the reactor’s biomass (Table 4) with values decreasing during the operation, suggesting a higher selective pressure in the membrane (Section 3.1.1).

**TABLE 4:** Microbiota alfa-diversity metrics (Neff Shannon and ASV richness) of the inoculum and samples of the bioreactors at the end of each operating phase.

	Neff Shannon	Richness
Inoculum	141	341
AnMBR phase I	182	418
AnMBR phase II	140	298
AnMBR phase III	136	339
AnMBR phase IV	114	300
AnMBR phase V	65	279
AnMBR phase VI	63	382
Membrane phase III	87	291
Membrane phase V	46	225
Membrane phase VI	24	177
UASB phase I	137	223
UASB phase II	168	388
UASB phase III	113	298
UASB phase IV	104	306
UASB phase V	101	315
UASB phase VI	97	292

**FIGURE 4:**
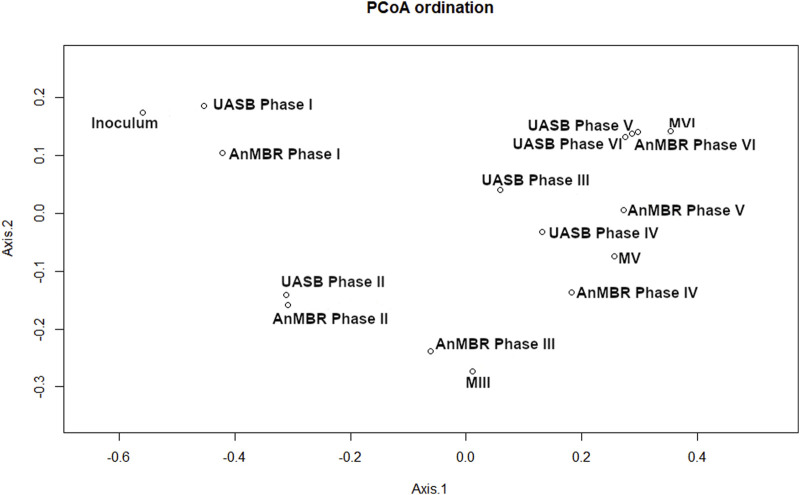
Beta diversity for the inoculum, biomass samples from the sludge of the AnMBR and UASB reactor at end of each operating phase (I-VI) and from the membrane (M) at the end of phases III, V and VI. PCoA of the Bray-curtis distance was performed with the 15 most abundant ASVs for each sample.

The beta diversity analysis (PCoA Bray-Curtis) presents the similarity of the communities by grouping similar samples. Figure 4 shows a cluster that was formed by the inoculum samples and both reactors in Phase I. The increasing distance from the inoculum to the clusters of the other samples demonstrate the phylogenetic distance between the microbial communities among the different operational stages. In general, the microbial communities of the UASB reactor and the AnMBR were overall similar with samples clustering together according to the operational phases, except for Phases III and V (Section 3.3.2; Section 3.3.3). These results suggest that operational conditions instead of configuration of the reactor influenced the selection of microorganisms. The analysis of the membrane-attached biomass and its comparison to the suspended biomass in the AnMBR show similarities between the microbial communities in both matrices.

#### 3.3.2 Microbial community analysis in the AnMBR

**FIGURE 5:**
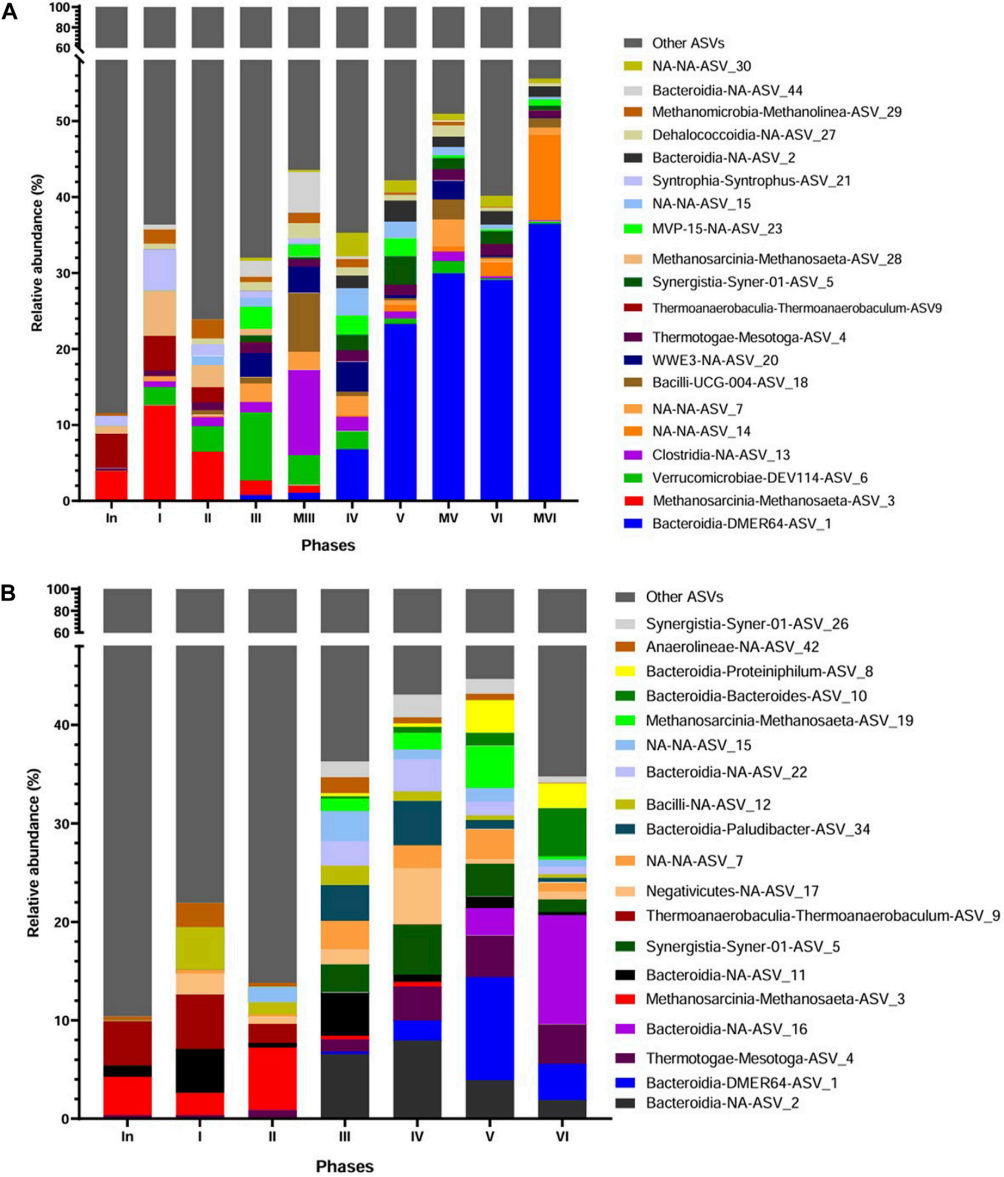
Taxonomic distribution according to the 16S rRNA gene amplicon sequencing analysis at Class-Genera level of the inoculum (In) and biomass samples from the sludge of the AnMBR at end of each operating phase (I-VI) and from the membrane (M) at the end of phases III, V and VI **(A)**, and biomass samples from the sludge of the UASB reactor **(B)**. NA-NA: ASVs not classified.

Figure 5A shows that the majority of amplicon sequence variant ASVs (85.6%) in the inoculum had a relative abundance (RA) of less than 1%, which increased during the operation of the AnMBR. The adaptation phase of the inoculum to vinasse (Phase I) promoted the selection of the acetoclastic methanogenic *Methanosaeta* ASV_3 (5.51%) followed by *Thermoanaerobaculum*-ASV_9 (4.50%) and in minor proportion by *Metholinea*-ASV_29 (0.7%), *Mesotoga*-ASV_4, *Verrucomicrobia*-DEV114-ASV_6 and *Bacteroidia-Paludibacter*-ASV_37 (sum of 1.22%), but still with predominance of ASVs lesser than 1%. The microbial community in Phase I efficiently converted the organic matter of diluted vinasse (Section 3.1; Table 3). The decrease in the HRT and consequently the increase in OLR from Phase II to III favored bacteria such as DEV114 (8.93%), WWE3-ASV_20 (3.12%), MVP15-ASV_23 (2.9%), *Paulidibacter* (2.55%) and *Bacteroidia*-NA-ASV_44 (2.1%) that peaked in Phase III, but negatively impacted the RA of the acetoclastic methanogen *Methanosaeta* ASV_3 with a decrease from 6.5% to 1.9%. Also, the hydrogenotrophic methanogen *Methanolinea* ASV_29 showed a decrease in its RA from 2.5% to 0.7%. In Phase III, the microbial community of the membrane cake layer was also analyzed showing that *Clostridia*-ASV_13, *Bacilli*-ASV_18, *Bacteroidia*-ASV_44 and *Methanolinea* were preferentially attached to the membrane. *Bacteroidia* encompass microorganisms that degrade complex organic compounds during hydrolysis and acidogenesis being part of the core microbiome in anaerobic reactors fed with vinasse (Siqueira et al., 2022; Callejas et al., 2022; Iltchenco et al., 2020; 2021; Pierangelli et al., 2024). *Clostridia* and *Bacilli* besides fermentation are also reported as potentially syntrophic acetate oxidizers (Mosbaek et al., 2016; Hao et al., 2021). The change of the membrane and increase in HRT in Phase IV resulted in minimal changes in abundance and composition of the microbial community in the reactor´s biomass, except by the increase in RA of DMER64-ASV1 from 0.75% to 6.81% (Figure 5A). The increase in the OLR in Phases V and VI was followed by a dominance of DMER64 (23.3%–29%). The other ASVs, such as, WWE3, *Clostridia*, DEV114, and *Methanolinea* were in minor RA (<1%). The membrane cake layer analyzed in Phases V and VI also showed a dominance of DMER64 with RA of 30% at Phase V and 36.5% at phase VI. DMER64 has been proposed as a) potential short volatile fatty acid (SVFA) producer, such as propionic and acetic acid (Ghosh et al., 2020; Zhang et al., 2022) and b) potential syntrophic bacteria that oxidize propionic and butyric acids via direct electron transfer (DIET) to methanogens (Lee et al., 2019; Ziganshina et al., 2020). The dominance of hydrolytic and fermentative microorganisms over methanogenic archaea, even with the selection of the potentially syntrophic DMER64, was observed in the same period in which decay of the performance of the reactor occurred (Phase VI) (Section 3.1; Table 3).

After Phase III, the removal of polyphenols was assessed by the phenol removal efficiency (REphenol) varying from 30% ± 17% (Phase VI) to 61% ± 11.5% (Phase IV) (Section 3.1; Table 3). Apart from ASVs of *Bacteroidetes* and *Dehalococcoidia* that are related to polyphenols and phenol degradation (Rastmanesh, 2011; Yu et al., 2023) no sound phenol or phenolic degraders were identified. Unknown ASVs (NAs) 7, 14, 30, WWE3, and MVP 15 were enriched from Phase III to Phase VI without a defined pattern (Figure 5A). However, a lack of information about these microorganisms hinders further understanding of their possible implication in the system, including polyphenols degradation.

#### 3.3.3 Microbial community analysis in the UASB reactor

The majority of ASVs (89.7%) (genus level) in the inoculum of the UASB reactor were in RA of less than 1% (Figure 5B), a result similar as the one observed in the inoculum of the AnMBR (Section 3.3.2). Few ASVs were in RA over 1%, e.g., *Methanosaeta*-ASV_3 (3.9%), *Thermoanaerobaculum*-ASV_9 (4.5%) and *Bacteroidia*-NA-ASV_11 (1.1%). During Phase I (adaptation to vinasse), the acetoclastic methanogenic *Methanosaeta* ASV_3 and the bacteria *Thermoanaerobaculum*-ASV_9 was barely affected with RA of 2.3% and 5.5%, respectively. The relative abundances of Bacteroidia-NA-ASV_11 increased from 1.15% to 4.5%, while Negativicutes-NA-ASV_17, Bacilli-NA-ASV_12, and Anaerolineae-NA-ASV_42, which were virtually absent in the inoculum, developed in Phase I with relative abundances of 2.1%, 4.3%, and 2.4%, respectively. In Phase II, with the increase of the OLR (Table 2), only the RA of *Methanosaeta* ASV_3 increased (2.3%–6.4%) while bacterial groups detected in the previous phase decreased in RA (sum of 4.3%). In Phases III and IV, *Bacteroidia* class was the most favored, with a sum of all ASVs of 17.8% and 19.5%, respectively. The methanogenic archaea *Methanosaeta*-ASV_3 was replaced by *Methanosaeta*-ASV_19 after Phase III, having similar abundance in Phases III and IV (1.4%–1.7%). The other microorganisms, however, were barely affected. In Phase V, with the increase in OLR but the same HRT of the previous phase, mainly DMER64 and *Methanosaeta* RA was favored, increasing from 2% to 10.5% and from 1.6% to 4.3%, respectively. In Phase VI, DMER64 dropped to 3.9%. Only *Bacteroides*-ASV-10, and *Bacteroidia*-NA-ASV_16 increased in RA from 1.3% to 4.9% and from 2.7% to 11%, respectively. The other ASVs related to Domain Bacteria also decreased in RA. Similarly, the methanogenic archaea *Methanosaeta* dropped from 4.3% to 0.33%. These results show that most probably the increase of OLR and the sulfide concentration in this phase (Section 3.1.3.) negatively affected the microbial community of the reactor.

#### 3.3.4 Abundant microbial species involved in sulfate reduction

Sulfate reduction in the AnMBR and UASB reactor was high with sulfate removal efficiencies higher than 81% and 73%, respectively (Section 3.1 Table 3). However, literature-reported sulfate reducing bacteria (SRB) were in low RA in both reactors. *Desulfovibrio* and *Desulfomicrobium* were the detected SRB in the AnMBR (Supplementary Table S1). *Desulfovibrio* was present in all the phases in both the sludge and in the membrane cake layer with a sum of ASVs varying between 0.1% and 0.67%, and *Desulfomicrobium* was present from Phase IV to VI with a higher abundance in the sludge (sum ASVs between 0.3% and 1.48%) than in the membrane-attached biomass (sum ASVs between 0.16% and 0.52%). The SRB detected in the UASB reactor were *Desulfitobacteria*, *Desulfovibrio*, and *Desulfobacteria-*Sva0081 (Supplementary Table S2). The ASVs related to *Desulfovibrio* were present in all phases with a sum varying between 0.2% and 1.44%, whereas Sva-0081 and *Desulfitobacteriia* were present in the inoculum up to Phase II with a sum of 0.41% and 0.39%, respectively. Strains of *Desulfovibrio* and *Desulfomicrobium* are able to use H_2,_ acetic, lactic and formic acid, pyruvate, and ethanol as carbon source and electron donors for sulfate reduction (Dias et al., 2008; Kuever et al., 2015). Based on SIP-experiments fed with marked acetate and sulfate, clades of Sva0081 reduced sulfate coupled with acetate oxidation (Wunder et al., 2021). Strains of *Desulfitobacteriia* use organic acids and H_2_ for sulfate reduction (Lai et al., 2020). Latic and acetic acid and ethanol were present in sugarcane vinasse (Table 1); therefore, its use by the sulfate reducers might be expected.

High sulfate reduction activity but low RA of sulfate reducers was previously reported by Hausmann et al. (2016) and Fuess et al. (2023). The former authors observed that most responsive operational taxonomic units related to SRB increased their rRNA content but only weakly increased in genomic abundance (DNA) over 50 days, which suggests a strategy of rare members to display activity compatible with the low genomic abundance. Decoupling between abundance and activity were shown in studies employing both DNA and RNA analyses simultaneously (De Vrieze et al., 2016; Guo et al., 2020; Pierangeli, et al., 2024).

A regularized canonical correlation analysis (rCCA) applied to address how operational and environmental parameters such as OLR, pH, organic acids effluent, %CH4 in biogas, removal efficiency of polyphenols, COD and sulfate, and the ASVs were related is presented in Supplementary Material.

## 4 Conclusion

This study compared the performance of AnMBR and UASB reactors treating sugarcane vinasse under varying operational conditions. The results demonstrated that the AnMBR is more effective than the UASB reactor for the treatment of vinasse, especially at an OLR of 6 g COD. L^-1^. d^-1^, achieving 88% ± 2% COD removal, 99% ± 0.6% sulfate removal, and 58% ± 15% polyphenol removal in comparison to the 75% ± 7% COD removal, 99% ± 0.5% sulfate removal, and 11% ± 2% polyphenol removal of the UASB reactor. The complete sludge retention in AnMBR contributed to superior effluent quality compared to the UASB reactor, and despite sludge degranulation, the AnMBR maintained its treatment efficiencies.

Both reactors exhibited sensitivity to increasing OLR, which affected the microbial community. In the AnMBR, dominant populations included *Clostridia, Bacteroidia, Mesotaga, Syner-01, Dehalococcoidia*, and *Bacteroidia*-DMER64, with a decline in *Methanolinea* and *Methanosaeta*. In contrast, the UASB reactor showed dominance of *Bacteroidia, Proteiniphilum, Syner-01, Bacteroidia*-DMER64, with a decline in *Methanosaeta*. Despite high sulfate reduction, the abundance of SRB was low, suggesting a decoupling of their presence from activity levels.

Further research is necessary to develop strategies for controlling fouling and enhancing volumetric organic loads and membrane flux, making the process more feasible for large-scale applications.

## Data Availability

The datasets presented in this study can be found in online repositories. The names of the repository/repositories and accession number(s) can be found in the article/Supplementary Material.

## Abbreviations

AD, Anaerobic digestion; ASV, Amplicon sequence variant; AnMBR, Anaerobic membrane bioreactor; CH, Carbohydrates; COD, Chemical oxygen demand; CODt, Total chemical oxygen demand; CODs, Soluble chemical oxygen demand; HRT, Hydraulic retention time; J, Flux; NA, Unknow microorganism; OLR, Volumetric organic loading rate; PSD, Particle size distribution; RA, Relative abundance; RE, Removal efficiency; SD, Standard deviation; SLR, Specific organic loading rate; TLS, Total liquid sulfide; TSS, Total suspended solids; UASB, Upflow anaerobic sludge blanket; UF, Ultrafiltration; VFA, Volatile fatty acids; VSS, Volatile suspended solids.

## References

[B1] Adorno M. A. T. Hirasawa J. S. Varesche M. B. A. (2014). Development and validation of two methods to quantify volatile acids (C2-C6) by GC/FID: headspace (automatic and manual) and liquid-liquid extraction (LLE). Am. J. Anal. Chem. 05 (07), 406–414. 10.4236/ajac.2014.57049

[B2] Albanez R. Lovato G. Zaiat M. Ratusznei S. M. Rodrigues J. A. D. (2016). Optimization, metabolic pathways modeling and scale-up estimative of an AnSBBR applied to biohydrogen production by co-digestion of vinasse and molasses. Int. J. Hydrogen Energy 41, 20473–20484. 10.1016/j.ijhydene.2016.08.145

[B3] Aquino S. Fuess L. T. Pires E. C. (2017). Media arrangement impacts cell growth in anaerobic fixed-bed reactors treating sugarcane vinasse: structured vs. randomic biomass immobilization. Bioresour. Technol. 235, 219–228. 10.1016/j.biortech.2017.03.120 28365350

[B4] Araujo M. N. Vargas S. R. Soares L. A. Trindade L. F. Fuess L. T. Adorno M. A. T. (2023). Rapid method for determination of biogas composition by gas chromatography coupled to a thermal conductivity detector (GC-TCD), International Journal of Environmental Analytical Chemistry, APHA, 2017. Standard Methods for the Examination of Water and Wastewater. Am. Public Heal. Assoc. Am. Water Work. Assoc. Water Environ. Fed. 23. 10.1080/03067319.2023.2210055

[B5] Bolger A. M. Lohse M. Usadel B. (2014). Trimmomatic: a flexible trimmer for Illumina sequence data. Bioinformatics 30 (15), 2114–2120. Epub 2014 Apr 1. PMID: 24695404; PMCID: PMC4103590. 10.1093/bioinformatics/btu170 24695404 PMC4103590

[B6] Callahan B. J. McMurdie P. J. Rosen M. J. Han A. W. Johnson A. J. A. Holmes S. P. (2016). DADA2: high-resolution sample inference from Illumina amplicon data. Nat. Methods 13 (7), 581–583. 10.1038/nmeth.3869 27214047 PMC4927377

[B7] Callejas C. López I. Bovio-Winkler P. Etchebehere C. Borzacconi L. (2022). Temporal analysis of the microbiota involved in the anaerobic degradation of sugarcane vinasse in a full-scale methanogenic UASB reactor. Biomass Convers. biorefin. 12, 3887–3897. 10.1007/s13399-021-01281-8

[B8] Camiloti P. R. Oliveira G. H. D. Zaiat M. (2016). Sulfur recovery from wastewater using a micro-aerobic external silicone membrane reactor (ESMR). Water Air Soil Pollut. 227, 31. 10.1007/s11270-015-2721-y

[B9] Cao K. Rohart F. Gonzalez I. Dejean S. Gautier B. Bartolo F. (2016). mixOmics: omics. Data integration project. R. package version 6 (1.1). 10.18129/B9.bioc.mixOmics

[B10] Chen Y. Cheng J. J. Creamer K. S. (2008). Inhibition of anaerobic digestion process: a review. Bioresour. Technol. 99, 4044–4064. 10.1016/j.biortech.2007.01.057 17399981

[B11] Contreras-Contreras J. A. Bernal-González M. Solís-Fuentes J. A. Del Carmen Durán-Domínguez-De-Bazúa M. (2020). Polyphenols from sugarcane vinasses, quantification, and removal using activated carbon after biochemical treatment in laboratory-scale thermophilic upflow anaerobic sludge blanket reactors. Water, Air, Soil Pollut. 231, 401. 10.1007/s11270-020-04733-5

[B12] Dacanal G. C. Feltre G. Thomazi M. G. Menegalli F. C. (2016). Effects of pulsating air flow in fluid bed agglomeration of starch particles. J. Food Eng. 181, 67–83. 10.1016/j.jfoodeng.2016.03.004

[B13] Dereli R. K. Ersahin M. E. Ozgun H. Ozturk I. Jeison D. Van Der Zee F. (2012). Potentials of anaerobic membrane bioreactors to overcome treatment limitations induced by industrial wastewaters. Bioresour. Technol. 122, 160–170. 10.1016/j.biortech.2012.05.139 22749827

[B14] De Vale R. J. (2021). A review of the factors affecting the performance of anaerobic membrane bioreactor and strategies to control membrane fouling. Rev. Environ. Sci. Biotechnol. 20, 607–644. 10.1007/s11157-021-09580-2

[B15] De Vrieze J. Regueiro L. Props R. Vilchez-Vargas R. Jáuregui R. Pieper D. H. (2016). Presence does not imply activity: DNA and RNA patterns differ in response to salt perturbation in anaerobic digestion. Biotechnol. Biofuels 9 (1), 244. 10.1186/s13068-016-0652-5 27843490 PMC5103597

[B16] Dias M. Salvado J. C. Monperrus M. Caumette P. Amouroux D. Duran R. (2008). Characterization of Desulfomicrobium salsuginis sp. nov. and Desulfomicrobium aestuarii sp. nov., two new sulfate-reducing bacteria isolated from the Adour estuary (French Atlantic coast) with specific mercury methylation potentials. Syst. Appl. Microbiol. 31 (1), 30–37. 10.1016/j.syapm.2007.09.002 18453046

[B17] Dvořák L. Gómez M. Dolina J. Cěrnín A. (2015). Anaerobic membrane bioreactors—a mini review with emphasis on industrial wastewater treatment: applications, limitations and perspectives. Desalination Water Treat. 57, 19062–19076. 10.1080/19443994.2015.1100879

[B18] Fuess L. T. Braga A. F. M. Eng F. Gregoracci G. B. Saia F. T. Zaiat M. (2023). Solving the bottlenecks of sugarcane vinasse biodigestion: impacts of temperature and substrate exchange on sulfate removal during dark fermentation. Chem. Eng. J. 455, 140965. 10.1016/j.cej.2022.140965

[B19] Fuess L. T. Colling B. Ferreira M. Cristina M. Ferreira A. Loureiro M. (2018). Diversifying the technological strategies for recovering bioenergy from the two-phase anaerobic digestion of sugarcane vinasse: an integrated techno-economic and environmental approach. Renew. Energy 122, 674–687. 10.1016/j.renene.2018.02.003

[B20] Fuess L. T. Garcia M. L. (2014). Implications of stillage land disposal: a critical review on the impacts of fertigation. J. Environ. Manage. 145, 210–229. 10.1016/j.jenvman.2014.07.003 25058869

[B21] Fuess L. T. Garcia M. L. (2015). Bioenergy from stillage anaerobic digestion to enhance the energy balance ratio of ethanol production. J. Environ. Manage. 162, 102–114. 10.1016/j.jenvman.2015.07.046 26233583

[B22] Galinha C. F. Sanches S. Crespo J. G. (2018). Membrane bioreactors, fundamental modeling of membrane systems: membrane and process performance, 209–249. Chap. 6.

[B23] Garcia Rea V. S. Egerland Bueno B. Cerqueda-García D. Muñoz Sierra J. D. Spanjers H. van Lier J. B. (2022). Degradation of p-cresol, resorcinol, and phenol in anaerobic membrane bioreactors under saline conditions. Chem. Eng. J. 430, 132672. 10.1016/j.cej.2021.132672

[B24] Ghosh P. Kumar M. Kapoor R. Kumar S. S. Singh L. Vijay V. (2020). Enhanced biogas production from municipal solid waste via co-digestion with sewage sludge and metabolic pathway analysis. Bioresour. Technol. 296, 122275. 10.1016/j.biortech.2019.122275 31683109

[B25] Guo H. van Lier J. B. de Kreuk M. (2020). Digestibility of waste aerobic granular sludge from a full-scale municipal wastewater treatment system. Water Res. 173 (February), 115617. 10.1016/j.watres.2020.115617 32070832

[B26] Hao Y. Hao S. Andersen-Nissen E. Mauck W. M. Zheng S. Butler A. (2021). Integrated analysis of multimodal single-cell data. Cell 184, 3573–3587.e29. 10.1016/j.cell.2021.04.048 34062119 PMC8238499

[B27] Hatano K. I. Kikuchi S. Miyakawa T. Tanokura M. Kubota K. (2008). Separation and characterization of the colored material from sugarcane molasses. Chemosphere 71 (9), 1730–1737. 10.1016/j.chemosphere.2007.12.019 18267325

[B28] Hausmann B. Knorr K. H. Schreck K. Tringe S. G. Glavina Del Rio T. Loy A. (2016). Consortia of low-abundance bacteria drive sulfate reduction-dependent degradation of fermentation products in peat soil microcosms. ISME Journ. 10 (10), 2365–2375. 10.1038/ismej.2016.42 PMC4930147 27015005

[B29] He X. Meng F. G. Lin A. L. Zhou Z. B. Chen Y. W. Tang C. Y. Y. (2015). Mono valention-mediated fouling propensity of model proteins during low-pressure membrane filtration. Sep. Purif. Technol. 152, 200–206. 10.1016/j.seppur.2015.08.003

[B30] Hoarau J. Caro Y. Grondin I. Petit T. (2018). Sugarcane vinasse processing: toward a status shift from waste to valuable resource: a review. Water Process Eng. 24, 11–25. 10.1016/j.jwpe.2018.05.003

[B31] Iltchenco J. Peruzzo V. Eva Magrini F. Marconatto L. Paula Torres A. Luiz Beal L. (2021). Microbiota profile in mesophilic biodigestion of sugarcane vinasse in batch reactors. Water Sci. Technol. J. Int. Assoc. Water Pollut. Res. 84, 2028–2039. 10.2166/wst.2021.375 34695028

[B32] Jiménez A. M. Borja R. Martín A. (2003). Aerobic-anaerobic biodegradation of beet molasses alcoholic fermentation wastewater. Process Biochem. 38 (9), 1275–1284. 10.1016/s0032-9592(02)00325-4

[B33] Klindworth A. Pruesse E. Schweer T. Peplies J. Quast C. Horn M. (2012). Evaluation of general 16S ribosomal RNA gene PCR primers for classical and next-generation sequencing-based diversity studies. Nucleic Acids Res. 41 (1), e1. 10.1093/nar/gks808 22933715 PMC3592464

[B34] Kuever J. Rainey F. A. Widdel F. (2015). “Desulfovibrionaceae fam. Nov,” in Bergey's manual of systematics of archaea and bacteria.

[B35] Lai R. Li Q. Cheng C. Shen H. Liu S. Luo Y. (2020). Bio-competitive exclusion of sulfate-reducing bacteria and its anticorrosion property. J. Petroleum Sci. Eng. 194, 107480. 10.1016/j.petrol.2020.107480

[B36] Le-Clech P. Chen V. Fane T. A. G. (2006). Fouling in membrane bioreactors used in wastewater treatment. J. Memb. Sci. 284, 17–53. 10.1016/j.memsci.2006.08.019

[B37] Lee J. Koo T. Yulisa A. Hwang S. (2019). Magnetite as an enhancer in methanogenic degradation of volatile fatty acids under ammonia-stressed condition. J. Environ. Manag. 241, 418–426. 10.1016/j.jenvman.2019.04.038 31035235

[B38] Lin H. Peng W. Zhang M. Chen J. Hong H. Zhang Y. (2013). A review on anaerobic membrane bioreactors: applications, membrane fouling and future perspectives. Desalination 314, 169–188. 10.1016/j.desal.2013.01.019

[B39] Magalhães N. C. Silva A. F. R. Cunha P. V. M. Drewes J. E. Amaral M. C. S. (2020). Role of nanofiltration or reverse osmosis integrated to ultrafiltration-anaerobic membrane bioreactor treating vinasse for the conservation of water and nutrients in the ethanol industry. J. Water Process Eng. 36, 101338. 10.1016/j.jwpe.2020.101338

[B40] Moraes B. S. Junqueira T. L. Pavanello L. G. Cavalett O. Mantelatto P. E. Bonomi A. (2014). Anaerobic digestion of vinasse from sugarcane biorefineries in Brazil from energy, environmental, and economic perspectives: profit or expense? Appl. Energy 113, 825–835. 10.1016/j.apenergy.2013.07.018

[B41] Mosbæk F. Kjeldal H. Mulat D. Albertsen M. Ward A. J. Feilberg A. (2016). Identification of syntrophic acetate-oxidizing bacteria in anaerobic digesters by combined protein-based stable isotope probing and metagenomics. ISME J. 10, 2405–2418. 10.1038/ismej.2016.39 27128991 PMC5030692

[B42] Mota V. T. Santos F. S. Amaral M. C. S. (2013). Two-stage anaerobic membrane bioreactor for the treatment of sugarcane vinasse: assessment on biological activity and filtration performance. Bioresour. Technol. 146, 494–503. 10.1016/j.biortech.2013.07.110 23958682

[B43] Muñoz Sierra J. D. Oosterkamp M. J. Wang W. Spanjers H. Van Lier J. B. (2019). Comparative performance of upflow anaerobic sludge blanket reactor and anaerobic membrane bioreactor treating phenolic wastewater: overcoming high salinity. Chem. Eng. J. 366, 480–490. 10.1016/j.cej.2019.02.097

[B44] Oksanen J. Blanchet F. G. Kindt R. Legendre P. Minchin P. R. O’Hara R. B. (2022). Vegan: community ecology package. R. package version 2, 6–4. 10.32614/CRAN.package.vegan

[B45] Ozgun H. Kaan R. Evren M. Kinaci C. Spanjers H. Lier J. B. V. (2013). A review of anaerobic membrane bioreactors for municipal wastewater treatment: integration options, limitations and expectations. Sep. Purif. Technol. 118, 89–104. 10.1016/j.seppur.2013.06.036

[B46] Parkin G. F. Lynch N. A. Kuo W. Keuren E. L. V. Bhattacharya S. K. (1990). Interaction between sulfate reducers and methanogens fed acetate and propionate. Res. J. Water Pollut. Control Fed. 62, 780–788. 10.2307/25043913

[B47] Parsaeea M. Kiania M. K. D. Karimi K. (2019). A review of biogas production from sugarcane vinasse. Sludge Bioenergy 122, 117–125. 10.1016/j.biombioe.2019.01.034

[B48] Pierangeli G. M. F. Gregoracci G. B. Del Nery V. Pozzi E. de Araujo Junior M. M. Damianovic M. H. R. Z. (2024). Long-term temporal dynamics of total and potentially active microbiota affect the biogas quality from the anaerobic digestion of vinasse in a pilot-scale hybrid anaerobic reactor. Bioresour. Technol. Rep. 26, 101822. 10.1016/j.biteb.2024.101822

[B49] Quast C. Pruesse E. Yilmaz P. Gerken J. Schweer T. Yarza P. (2013). The SILVA ribosomal RNA gene database project: improved data processing and web-based tools. Nucleic Acids Res. 41 (D1), D590–D596. 10.1093/nar/gks1219 23193283 PMC3531112

[B50] Russ J. C. (2011). The image processing handbook. 4th ed. Boca Raton: CRC Press. 10.1201/9781420040760

[B51] Santos F. S. Ricci B. C. França Neta L. S. Amaral M. C. S. (2017). Sugarcane vinasse treatment by two-stage anaerobic membrane bioreactor: effect of hydraulic retention time on changes in efficiency, biogas production and membrane fouling. Bioresour. Technol. 245, 342–350. 10.1016/j.biortech.2017.08.126 28898829

[B52] Santos S. C. Rosa P. R. F. Sakamoto I. K. Varesche M. B. A. Silva E. L. (2014). Organic loading rate impact on biohydrogen production and microbial communities at anaerobic fluidized thermophilic bed reactors treating sugarcane stillage. Bioresour. Technol. 159, 55–63. 10.1016/j.biortech.2014.02.051 24632626

[B53] Seidel A. Elimelech M. (2002). Coupling between chemical and physical interactions innatural organic matter (NOM) fouling of nanofiltration membranes: implications for fouling control. J. Membr. Sci. 203, 245–255. 10.1016/S0376-7388(02)00013-3

[B54] Silva A. F. R. Magalhães N. C. Cunha P. V. M. Amaral M. C. S. Koch K. (2020). Influence of COD/SO42− ratio on vinasse treatment performance by two-stage anaerobic membrane bioreactor. J. Environ. Manage. 259, 110034. 10.1016/j.jenvman.2019.110034 31932266

[B55] Siqueira J. C. de Assemany P. Siniscalchi L. A. B. (2022). Microbial dynamics and methanogenic potential of co-digestion of sugarcane vinasse and dairy secondary effluent in an upflow anaerobic sludge blanket reactor. Bioresour. Technol. 361, 127654. 10.1016/j.biortech.2022.127654 35868464

[B56] Smith A. L. Stadler L. B. Love N. G. Skerlos S. J. Raskin L. (2012). Perspectives on anaerobic membrane bioreactor treatment of domestic wastewater: a critical review. Bioresour. Technol. 122, 149–159. 10.1016/j.biortech.2012.04.055 22608937

[B57] Van Haandel A. Van Lier J. B. (2015). “Role of anaerobic digestion in increasing the energy efficiency and energy output of sugar cane distilleries,” in Anaerobic biotechnology; environmental protection and resource recovery. Editors Fang H. H. P. Zhang T. (London, UK: World Scientific, Imperial College Press). Chapter 15.

[B58] Van Haandel A. C. (2005). Integrated energy production and reduction of the environmental impact at alcohol distillery plants. Water Sci. Technol. 52, 49–57. 10.2166/wst.2005.0497 16180408

[B59] Van Lier J. B. Mahmoud N. Zeeman G. (2020). “Anaerobic wastewater treatment,” in Biological wastewater treatment, principles, modelling and design. Editors Chen G. van Loosdrecht M. C. M. Ekama G. A. Brdjanovic D. 2nd Edition (London, UK: IWA Publishing), 701–756. Chapter 16, ISBN: 9781789060355.

[B60] Wang L. L. Wang L. F. Ye X. D. Yu H. Q. (2013). Hydration interactions and stability of soluble microbial products in aqueous solutions. Water Res. 47 (5), 5921–5929. 10.1016/j.watres.2013.07.014 23911223

[B61] Wunder L. C. Aromokeye D. A. Yin X. Richter-Heitmann T. Willis-Poratti G. Schnakenberg A. (2021). Iron and sulfate reduction structure microbial communities in (sub-)Antarctic sediments. ISME J. 15 (12), 3587–3604. 10.1038/s41396-021-01014-9 34155335 PMC8630232

[B62] Zhang Q. Wu L. Huang J. Qu Y. Pan Y. Liu L. (2022). Recovering short-chain fatty acids from waste sludge via biocarriers and microfiltration enhanced anaerobic fermentation. Resour. Conservation Recycl. 182, 106342. 10.1016/j.resconrec.2022.106342

[B63] Ziganshina E. E. Belostotskiy D. E. Bulynina S. S. Ziganshin A. M. (2020). Influence of granular activated carbon on anaerobic co-digestion of sugar beet pulp and distillers grains with solubles. Processes 8 (10), 1226–1316. 10.3390/pr8101226

