# Supplementary material for: Treatment of sugarcane vinasse in AnMBR and UASB: process performance and microbial community comparison
Source: Front Bioeng Biotechnol. 2024 Nov 8;12:1489807. doi: 10.3389/fbioe.2024.1489807 (PMC11600140; doi:10.3389/fbioe.2024.1489807)
Supplement: Supplementary file 1 [file DataSheet1.PDF]

Supplementary Material

S1 Volatile Fatty Acids Production

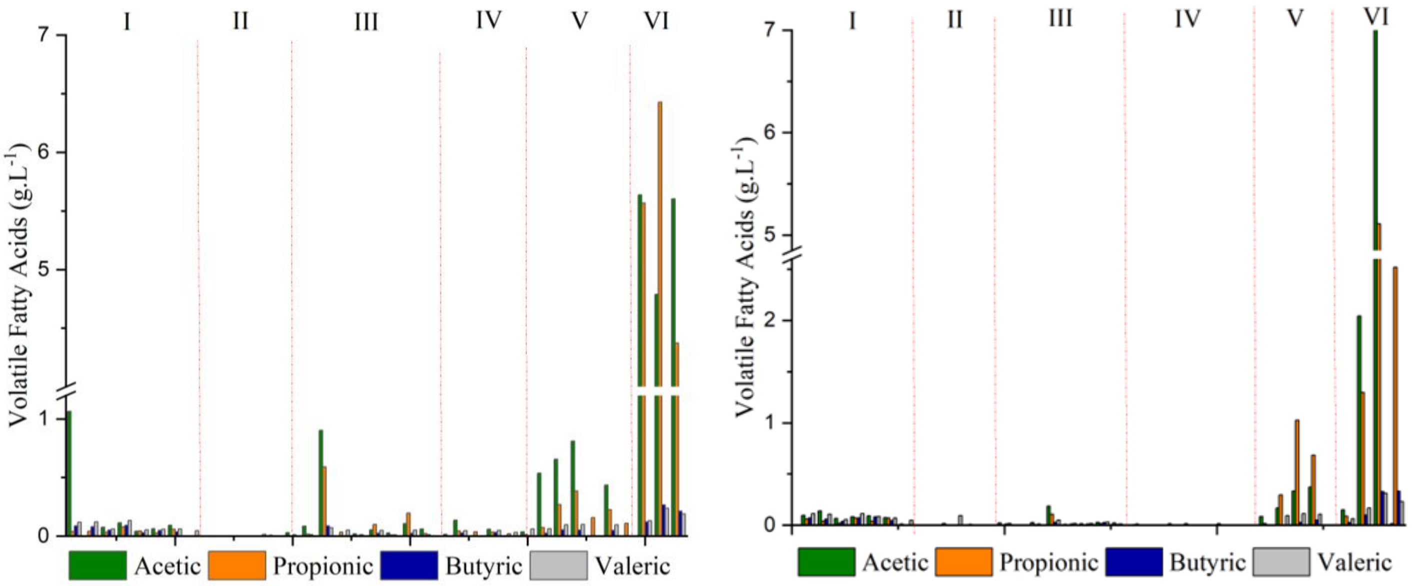

A **B**

Figure S1. Reactors monitoring over time of VFA accumulation (**A**) AnMBR (**B**) UASB.

S2 Carbohydrates and Sulphate Removal

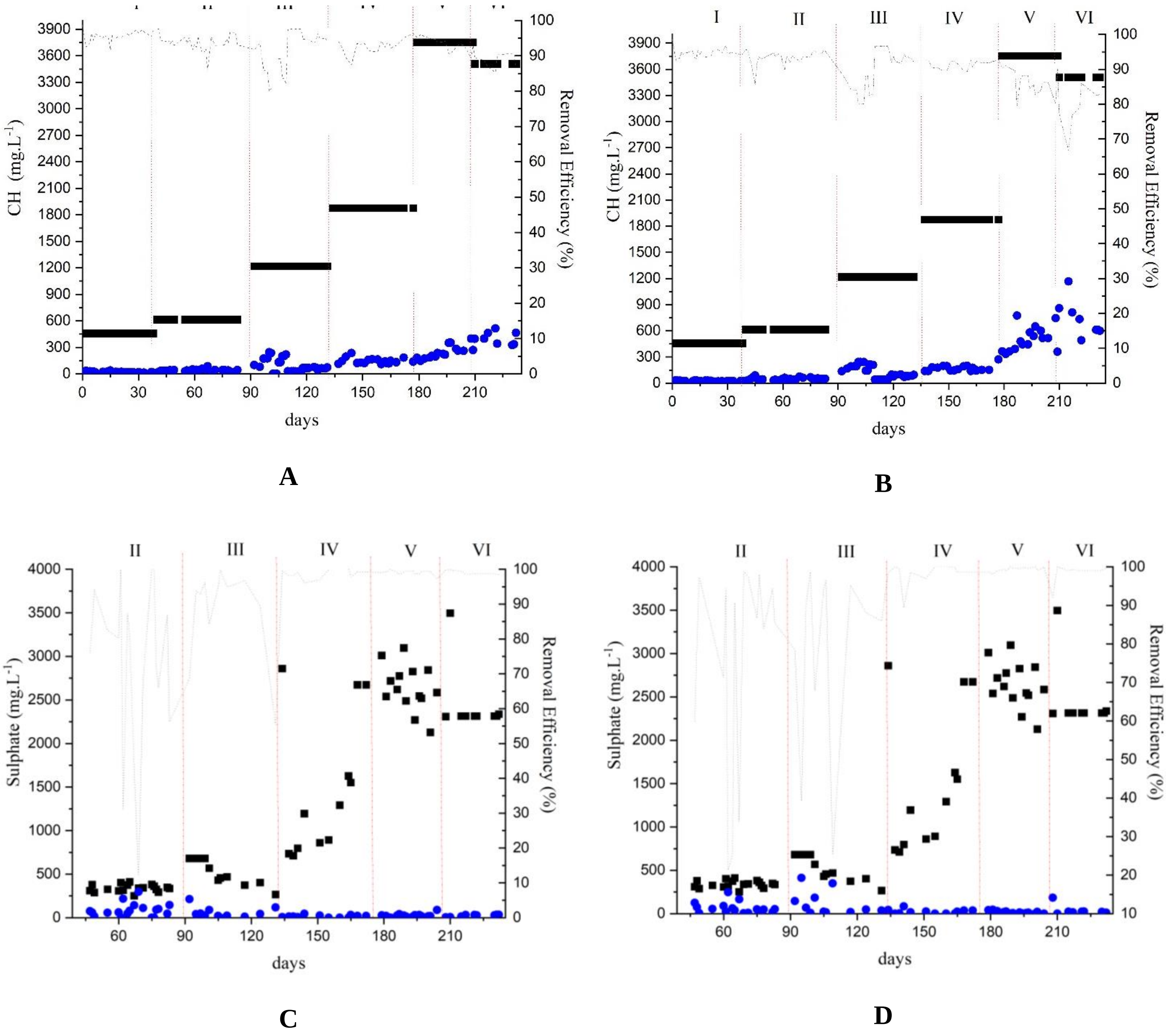

Figure S2. Reactors monitoring over time of carbohydrates (**A**) AnMBR (**B**) UASB, sulphate(**C**) AnMBR (**D**) UASB: (■) Feed (●) Effluent (---) Removal efficiency.

S3 Polyphenols Removal

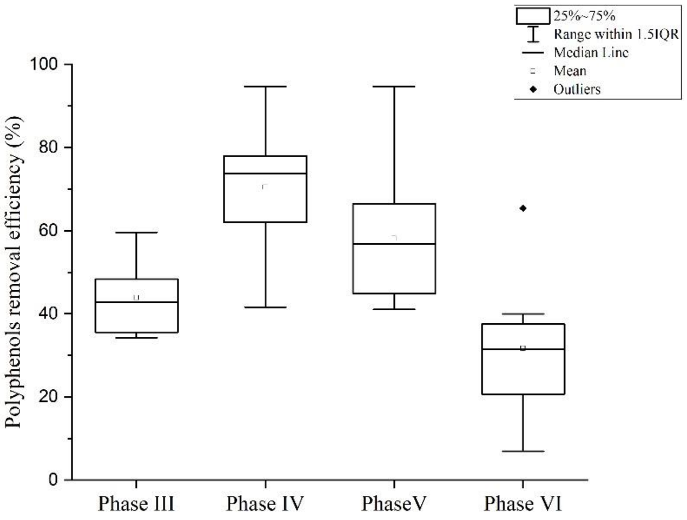

A

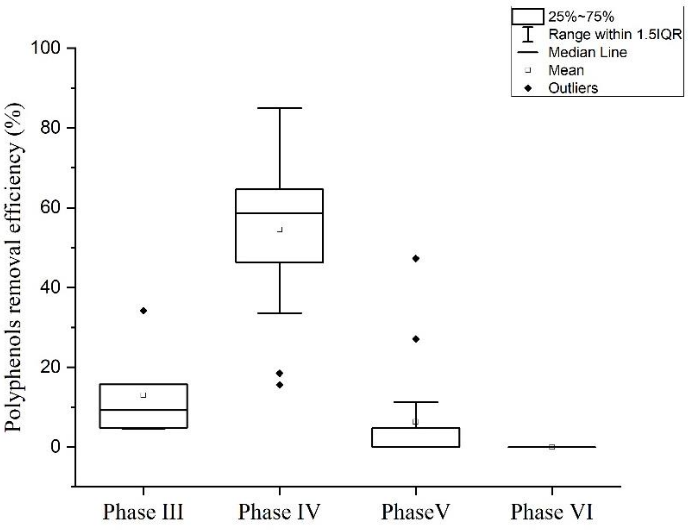

B

Figure S3. Reactors monitoring over time of polyphenols removal efficiency (**A**) AnMBR (**B**) UASB.

S4 Microbial community dynamics

At Phylum-Class level *Bacteroidia*, *Firmicutes* (*Clostridia* and *Bacilli* classes) followed by *Verrucomicrobiota*, *Synergistota* and *Chloroflexi* were present in the inoculum and prevailed during the operation of the AnMBR while relative abundance (RA) of unknown ASVs (NA-NA) decreased (Figure S4A). These Bacterial phyla are composed by microorganisms with hydrolytic, fermentative, and syntrophic capabilities, being commonly found in anaerobic digestion or co-digestion of vinasse and sugarcane leaf (Callejas et al. 2022; Adarme et al., 2022; Pierangelli et al., 2024, Sitthikitpanya et al., 2024). Regarding methanogenic archaea *Methosarcinia* and *Methanomicrobia* were present in the inoculum and throughout the operation (Figure S4A). As observed in the AnMBR, *Bacteroidia*, *Firmicutes* (*Clostridia*, *Bacilli* and *Negativicutes* classes) followed by *Verrucomicrobiota*, *Synergistota* and *Chloroflexi* were present in the inoculum and prevailed during the operation of the UASB reactor while RA of unknown ASVs (NA-NA) decreased (Figure S4B). Regarding methanogenic archaea also *Methosarcinia* and *Methanomicrobia* were present in the inoculum and throughout the operation (Figure S4B).

Figure S4. Taxonomic distribution according to the 16S rRNA gene amplicon sequencing analysis at Phyla-Class level of AnMBR (**A**) and UASB reactor (**B**) of the inoculum (In) and biomass samples from the sludge collected in the AnMBR at end of each operating phase (I-VI) and from the membrane (M) at the end of phases III, V and VI. NA-NA: ASVs not classified.

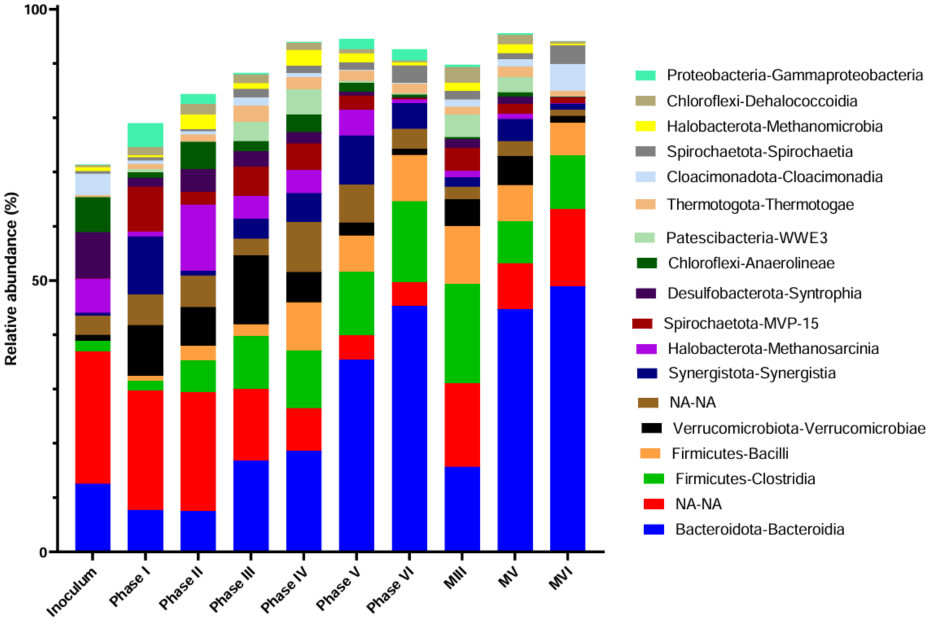

A

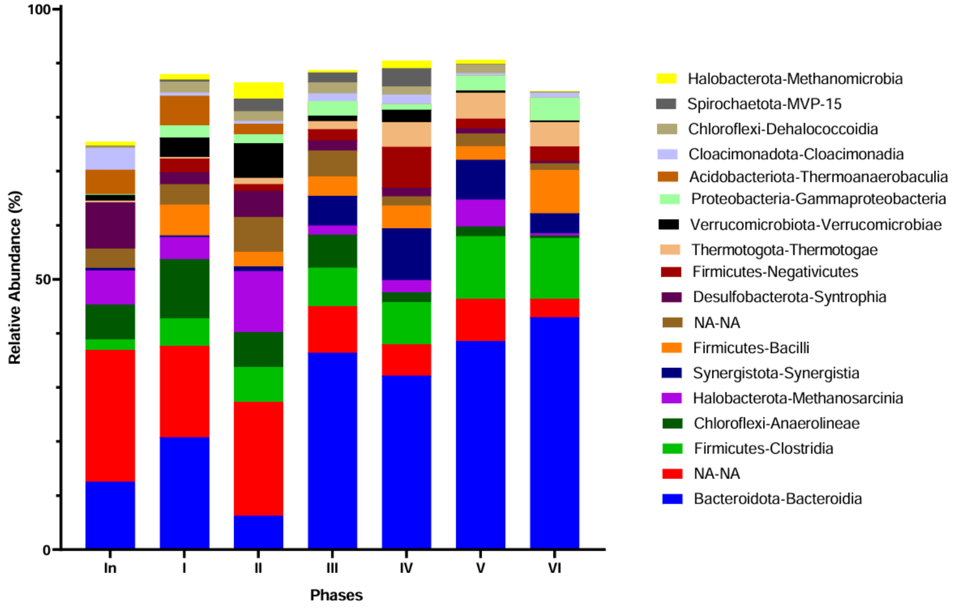

B

Table S1. Relative abundance (%) of ASVs related to sulfate reducing bacteria obtained from the inoculum and from the sludge of the AnMBR at the end of each operating phase and from the membrane (M) at the end of phases III, V and VI.

Class-Genera level	In	I	II	III	IV	V	VI	MIII	MV	MVI
Desulfovibrionia-Desulfovibrio ASV_152	0	0.5	0	0.21	0	0	0	0.21	0	0
Desulfovibrionia-Desulfovibrio ASV_258	0	0	0.26	0.23	0	0	0	0.23	0.07	0
Desulfovibrionia-Desulfovibrio ASV_58	0	0	0	0	0	0.21	0.34	0	0	0.1
Desulfovibrionia-Desulfovibrio ASV_372	0	0	0	0.23	0.17	0.09	0	0	0.12	0
Desulfovibrionia-Desulfovibrio-ASV_645	0	0	0	0	0	0	0	0.11	0	0
*Desulfovibrionia-Desulfomicrobium-*ASV_735	0	0	0	0	0.09	0.15	0	0	0.03	0
DesulfovibrioniaDesulfomicrobium ASV_78	0	0	0	0	0.21	1.06	1.48	0	0.49	0.16

Table S2. Relative abundance (%) of ASVs related to sulfate reducing bacteria obtained from the inoculum and from the sludge of UASB reactor at the end of each operating phase (I-VI)

Class-Genera level	In	I	II	III	IV	V	VI
Desulfovibrionia-Desulfovibrio ASV_152	0	1.06	0.07	0.25	0.12	0.09	0
Desulfovibrionia-Desulfovibrio ASV_258	0	0.26	0.16	0	0.21	0.07	0
Desulfovibrionia-Desulfovibrio ASV_58	0	0	0	0	0	0.59	1.44
Desulfobacteria-Sva0081 sediment group-ASV_339	0.10	0.06	0.25	0	0	0	0
Desulfitobacteriia-TC1-ASV_337	0.14	0.12	0.12	0	0	0.01	0

Regularized Canonical Correspondence Analysis

A regularized canonical correlation analysis (rCCA) was applied to address how operational and environmental parameters such OLR, pH, organic acids effluent, %CH4 in biogas, removal efficiency of polyphenols, COD and sulfate, and the ASVs were related. Figure S5A shows *Bacteroidia*-DMER64-ASV_1, *Bacteroidia-Proteiniphilum*-ASV_8, and *Verrucomicrobiae*-ASV_47 positively correlated with the increase in the OLR and acetic and propionic acids in AnMBR effluent, showing that these microorganisms were favored by the increase of organic matter with production of organic acids. DEV114-ASV_6, *Bacteroidia-Paludibacter*-ASV_37 and *Dehalococcoidia*-ASV_27 were strongly related with COD removal. *Methanolinea*-ASV_29 and WWE3-ASV_20 were positively correlated with %CH4 in biogas. Syner-01-ASV_5 was positively related to sulfate removal and butyric acid effluent.

The rCCA (Figure S5B) showed that the OLR and propionic and acetic acid in the UASB reactor effluent were strongly correlated with *Bacteroidia-Proteiniphilum*-ASV_8, *Bacteroidia*-NA-ASV_16, *Bacteroidia*-DMER64-ASV_1 and *Methanosaeta*-ASV_19. These results suggest that these microorganisms were favored by the increase of organic matter, and ASVs related to *Bacteroidia* were correlated with production of organic acids. It is interesting to note the strong correlation between DMER64-ASV_1 and *Methanosaeta*-ASV_19, suggesting the occurrence of syntrophic association between these microorganisms. *Mesotoga*-ASV_4 was correlated with butyric acid in the effluent and sulfate reduction. In fact, strains of *Mesotoga* are capable of fermentative metabolism producing organic acids as well as of oxidation of sugar in association with hydrogenotrophic sulfate reducers (Fadhlaoui et al., 2018). *Bacteroidia* ASVs 2 and 22 were strongly correlated with polyphenols removal. *Methanosaeta*-ASV_3 and *Paludibacter*-ASV_34 were positively correlated with COD and polyphenol removal. *Paludibacter* probably indirectly by the fermentation of sugarcane vinasse, and *Methanosaeta* by acetoclastic methanogenesis. The ASVs used in rCCA analysis are presented in Table S3 and Table S4.

Figure S5. Regularized Canonical Correspondence Analysis (rCCA) carried out with operating parameters, physical-chemical and microbial data obtained from the AnMBR (A) and UASB reactor (B) ASVs are represented by letter V with the number correspondent.

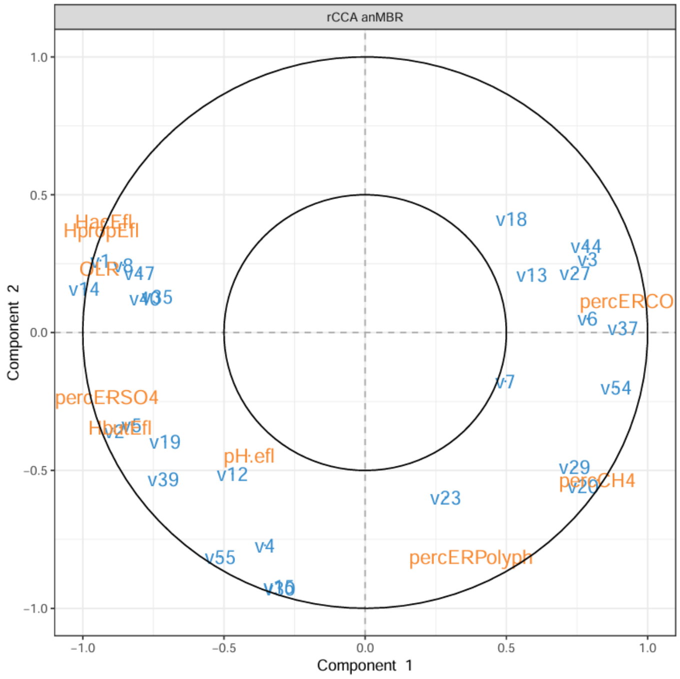

A

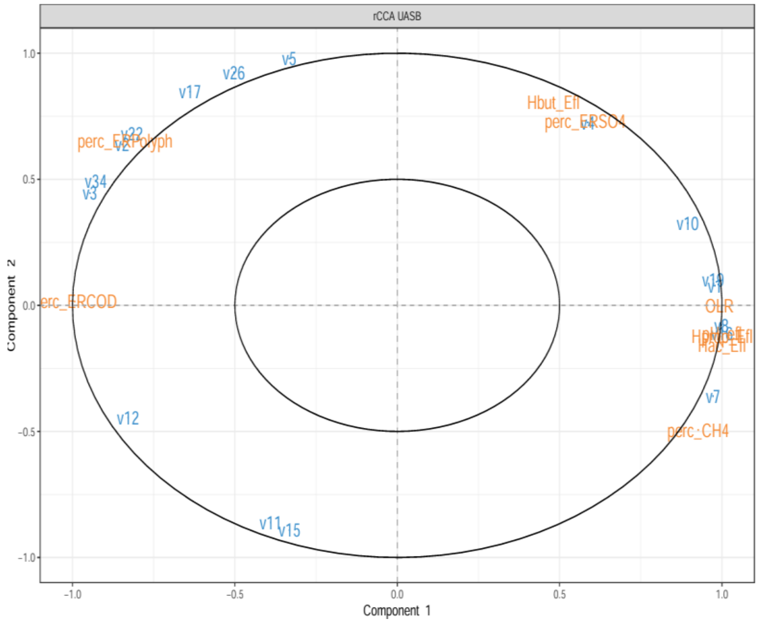

B

Table S3. ASVs of the UASB reactor used in rCCA analysis.

domain	phylum	genus	ASVs	Code	I	II	III	IV	V	VI
Bacteria	Bacteroidota	NA	ASV_2	v2	0	0	2605	3770	1655	778
Bacteria	Bacteroidota	DMER64	ASV_1	v1	0	0	82	949	4452	1493
Bacteria	Thermotogota	Mesotoga	ASV_4	v4	143	367	491	1619	1796	1644
Bacteria	Bacteroidota	NA	ASV_16	v16	0	0	0	0	1166	4506
Bacteria	Synergistota	Syner-01	ASV_5	v5	0	0	1127	2418	1406	538
Bacteria	Firmicutes	NA	ASV_17	v17	889	285	605	2704	215	324
Bacteria	Bacteroidota	NA	ASV_11	v11	1873	189	1731	356	508	130
NA	NA	NA	ASV_7	v7	164	102	1150	1104	1278	368
Bacteria	Bacteroidota	Paludibacter	ASV_34	v34	0	0	1435	2115	399	184
Bacteria	Firmicutes	NA	ASV_12	v12	1821	513	790	470	199	160
Archaea	Halobacterota	Methanosaeta	ASV_3	v3	954	2611	149	229	0	0
Bacteria	Bacteroidota	NA	ASV_22	v22	0	0	977	1549	594	321
Archaea	Halobacterota	Methanosaeta	ASV_19	v19	0	0	510	799	1813	134
Bacteria	NA	NA	ASV_15	v15	0	634	1207	458	588	292
Bacteria	Acidobacteriota	Thermoanaerobaculum	ASV_9	v9	2322	786	0	0	0	0
Bacteria	Bacteroidota	Bacteroides	ASV_10	v10	0	0	92	292	558	1997
Bacteria	Bacteroidota	Proteiniphilum	ASV_8	v8	0	0	128	175	1411	1024
Bacteria	Synergistota	Syner-01	ASV_26	v26	0	0	635	1096	645	254

Table S4. ASVs of the AnMBR used in rCCA analysis.

domain	phylum	genus	ASVs	Code	I	II	III	IV	V	VI	M III	M V	M VI
Bacteria	Bacteroidota	DMER64	ASV_1	v1	0	0	326	3200	9688	28138	431	11072	15102
Bacteria	Verrucomicrobiota	DEV114	ASV_6	v6	154	907	3864	1109	306	193	1611	593	129
Bacteria	Firmicutes	NA	ASV_13	v13	48	330	581	880	381	249	4605	466	66
NA	NA	NA	ASV_14	v14	0	0	0	96	346	1866	0	244	4640
Bacteria	Patescibacteria	NA	ASV_20	v20	0	0	1349	1840	153	194	1415	918	34
Bacteria	Firmicutes	UCG-004	ASV_18	v18	0	167	361	270	122	200	3176	973	518
NA	NA	NA	ASV_7	v7	45	74	1070	1208	258	412	977	1314	407
Bacteria	Synergistota	Syner-01	ASV_5	v5	0	0	404	964	1552	1676	134	558	247
Bacteria	Bacteroidota	Proteiniphilum	ASV_8	v8	0	0	0	14	325	4198	24	91	773
Bacteria	Thermotogota	Mesotoga	ASV_4	v4	47	290	583	714	594	1441	397	550	383
Bacteria	Bacteroidota	NA	ASV_2	v2	0	0	20	794	1167	1726	29	507	587
Bacteria	Spirochaetota	NA	ASV_23	v23	0	0	1252	1174	985	198	656	119	353
Bacteria	NA	NA	ASV_15	v15	0	323	508	1694	888	620	81	419	121
Bacteria	NA	NA	ASV_30	v30	0	0	187	1462	652	1418	116	331	252
Archaea	Halobacterota	Methanosaeta	ASV_3	v3	806	1769	852	0	0	0	416	0	0
Bacteria	NA	NA	ASV_39	v39	0	40	66	929	1214	1080	0	190	107
Bacteria	Spirochaetota	Sphaerochaeta	ASV_35	v35	0	0	0	0	213	2252	0	0	1157
Bacteria	Chloroflexi	NA	ASV_27	v27	49	219	513	505	321	383	808	542	178
Bacteria	Verrucomicrobiota	NA	ASV_47	v47	0	0	0	352	625	849	0	1192	397
Bacteria	Bacteroidota	NA	ASV_44	v44	41	0	895	144	17	14	2213	56	0
Bacteria	Firmicutes	NA	ASV_12	v12	106	330	230	285	341	1639	170	153	121
Bacteria	Bacteroidota	NA	ASV_40	v40	0	0	189	322	433	1095	0	737	575
Bacteria	Bacteroidota	Paludibacter	ASV_37	v37	0	192	1105	551	62	65	726	456	76

Archaea	Halobacterota	Methanosaeta	ASV_19	v19	0	39	284	726	1234	447	0	172	50
Bacteria	Firmicutes	Trichococcus	ASV_55	v55	0	0	0	1126	622	502	0	371	0
Archaea	Halobacterota	Methanolinea	ASV_29	v29	120	672	301	521	127	147	556	171	0
Bacteria	Spirochaetota	NA	ASV_54	v54	0	0	543	623	110	58	804	401	72
